# Health status and disease prevalences in French bulldogs in Germany: insights from a survey-based study

**DOI:** 10.1186/s40575-025-00149-8

**Published:** 2025-10-31

**Authors:** Mara A. G. Hinze, Konstantin O. H. Meyer, Romy M. Heilmann, Uwe Müller

**Affiliations:** 1 Institute of Immunulogy, Faculty of Veterinary Medicine, Leipzig University, Deutscher Platz 5, Leipzig, 04103 Germany; 2 School of Management, Technical University of Munich, Arcisstraße 21, Munich, 80333 Germany; 3 Department for Small Animals, Faculty of Veterinary Medicine, Leipzig University, An den Tierkliniken 23, Leipzig, 04103 Germany

**Keywords:** Allergy, Questionnaire, Hypersensitivity, Cancer, Cryptorchidism, Dystocia, Breeding, Purebred, Brachycephalic, Robinow-like syndrome, BOAS

## Abstract

**Background:**

The French bulldog has recently become one of the most popular dog breeds in the UK, Germany, and the USA. Known for its brachycephalic facial structure, characterized by a short muzzle and flat nose, the breed is predisposed to brachycephalic obstructive airway syndrome (BOAS) and other health concerns, including gastrointestinal, dermatological, and orthopedic conditions. These conditions are distressing for affected dogs and present significant challenges for their owners and healthcare providers. A survey targeting French bulldog owners in Germany was conducted to investigate the general health and prevalence of health problems in French bulldogs. Owners provided general information about their dogs, including sex, coat color, weight, height, neuter status, and overall health. Additionally, detailed health histories focused on specific organ systems—such as respiratory, dermatological, orthopedic, neurological, gastrointestinal, ocular, and behavioral conditions—were collected.

**Results:**

Responses were obtained for 574 French bulldogs, 499 of which were alive and 75 deceased at the time of data collection. Of these dogs, 49% were female and 51% male; the median age was 4 years. The most commonly reported abnormalities or diseases included tail malformations (98%), diagnosed or presumed hypersensitivities/allergies (52%), otitis externa/media (36%), stenotic nares (35%), pseudopregnancy (33%), dystocia (30%), elongated soft palate (26%), hemivertebrae (25%), conjunctivitis (22%), underbite (21%), *Giardia* spp. detection in fecal samples (20%), skin fold dermatitis (20%), dental calculus (19%), and intervertebral disc herniation (18%). Having a shorter nose (Fig. 3 a + b) was significantly associated with an increased susceptibility to conditions such as hypoplastic trachea, hemivertebrae, intervertebral disc disease (IVDD), and breathing abnormalities, including snoring, persistent respiratory noises, heat intolerance, and episodes of dyspnea. Among deceased dogs, the leading cause of death was cancer (47%), with various tumor types diagnosed. The average age at death was 8.3 years.

**Conclusion:**

This study reveals that a significant proportion of French bulldogs is affected by conditions involving the skin, respiratory system, and gastrointestinal tract. These findings highlight that efforts for ethical breeding practices, owner education, and breeding regulations continue to be critical for improving the health of the French bulldog breed. Such measures are an important means to reduce disease prevalence and improve the long-term health and welfare of the breed.

**Supplementary Information:**

The online version contains supplementary material available at 10.1186/s40575-025-00149-8.

## Background

The French bulldog originated in England from the Molossians, a breed of working dogs called *Bullenbeissers* [1]. After miniaturization following the prohibition of their original work, these small bulldogs were brought to France during the French Revolution, where they became popular among artists and fashion designers [1, 2]. The modern French bulldog was recognized by the French Kennel Club in 1898 [3].

The French bulldog, as a brachycephalic breed, is characterized by a short muzzle and a flat profile of the nose [3, 4]. Its small, compact, and muscular body, short tail, and bat ears provide a unique appearance. The cheerful, playful, and open character makes the French bulldog a popular family dog and companion [5]. These characteristics presumably contributed to the rapid increase in the number of French bulldogs in the USA, England, and Germany over the last years [5–8]. From 2014 to 2021 (over 7 years), the registrations of French bulldogs in the UK rose sixfold from 9,000 to over 54,000 new registrations annually [8]. The number of French bulldog registrations in Germany is recorded on a voluntary basis through the online platform TASSO [9]. However, since registration is not mandatory, the actual French bulldog population is likely larger. TASSO documented a rise in the number of French bulldogs from 10,848 annual registrations in 2017 to 13,657 registrations in 2020 (Lisa Frankenberger, personal communication, 19 September 2023). In 2022, the French bulldog was the most popular dog breed in the USA, and it is among the most popular purebred dog breeds in Germany and England [5, 6, 10]. However, recent UK Kennel Club data indicate a marked decline in new registrations of French bulldogs, as evidenced by a 51% decrease between 2021 and 2023 [8].

Given the breed characteristics, the number of French bulldogs worldwide has – from a healthcare provider’s perspective – shifted the focus from the cute appearance of the dogs to some concerning health problems. At the center of attention are respiratory conditions, collectively known as brachycephalic obstructive airway syndrome (BOAS) [11]. 70% of French bulldogs show varying signs of the BOAS complex, which can include an increased sensitivity to higher temperatures, exercise intolerance, and shortness of breath or dyspnea [12]. Causes for the clinical signs of BOAS are stenotic nares, an elongated soft palate, everted laryngeal saccules on the lateral aspects of the vestibular folds, narrowed rima glottidis, and/or collapse of the larynx and trachea [13]. Brachycephaly can be associated with Robinow-like syndrome, a genetic variation linked to malformation of skeletal structures, including the tail [14]. Shortness of the skull and spine and tail abnormalities are part of the Robinow-like syndrome and can lead to disorders frequently seen in French bulldogs, such as wedge-shaped vertebrae (hemivertebrae) and a truncated or kinked tail (screw tail) [15].

Besides the respiratory signs associated with BOAS, French bulldogs are reported to have an increased risk for other common disorders when compared to other dog breeds [16, 17]. Narrowing of the ear canal resulting from the characteristic skull formation carries a 14-fold increased risk for aural discharge when compared to the general dog population [17, 18]. Superfluous skin in the head and tail areas increases the risk of skin fold dermatitis by about 11-fold [17, 19]. Dystocia due to a narrowing in the skeletal birth canal is 9 times more likely to occur than in other dog breeds, and the risk of food hypersensitivity is about 7 times higher [4, 17]. With an average life expectancy of 4.5 years, characterized by a bimodal distribution, the French bulldog is one of the shortest-lived in comparison to other dog breeds [20].

Despite decades of research on the respiratory issues in brachycephalic dogs pursued since the 1980s and the general awareness of the breed’s health issues being linked to significant welfare concerns, the French bulldog remains a highly popular breed [7, 11, 21]. This might reflect that ownership of French bulldogs is chosen based on the breed’s distinctive appearance and personality rather than its overall health status [22, 23]. Specifically, the infantile facial appearance and joyful character seem to appeal to owners, whereas the breed-associated health problems in brachycephalic dogs are frequently underestimated by their owners [23, 24]. In addition, breed standards can pose restrictions on improving the general characteristics of any breed. In French bulldogs, the brachycephalic face with a very short nose and prognathism, as well as the short tail, are desired breed-specific features [25]. An inbreeding coefficient of 26% can restrict breeding to healthy offspring [4].

Some countries have implemented rules for the breeding of certain dog breeds if their phenotypic characteristics violate the terms of the national animal welfare law [26, 27]. The Netherlands, for example, introduced a traffic light-like warning system in 2019 to contain the breeding of brachycephalic dogs (e.g., dogs with a nose length of less than a third of the skull length are not allowed to be bred) [28]. The 2024 amendment in the national Animal Protection law in Germany also proposes restrictions on the breeding of dogs with inherited conditions, including respiratory conditions, exophthalmos, and dystocia [29].

In recent years, there has been a significant increase in awareness of the health issues associated with the French bulldog breed, prompting a critical need for further research and evaluation [17, 23]. Thus, the primary aim of this survey-based cross-sectional study was to provide a comprehensive analysis and update of the current overall health status of French bulldogs in a defined geographic region, focused on Germany, from April to June 2024. This research aims to inform breeders, veterinarians, and owners, fostering a sensible approach to the health care and breeding of French bulldogs and contributing to an improvement of the breed’s overall well-being and quality of life.

## Materials and methods

Based on the existing literature and the known breed-associated health concerns, this study hypothesizes that certain diseases are more common in the French bulldog than the overall dog population and may be linked to specific physical traits of these dogs. This study provides a descriptive and quantitative analysis based on a standardized, specifically designed survey targeting owners of pedigree French bulldogs.

**Fig. 1:**
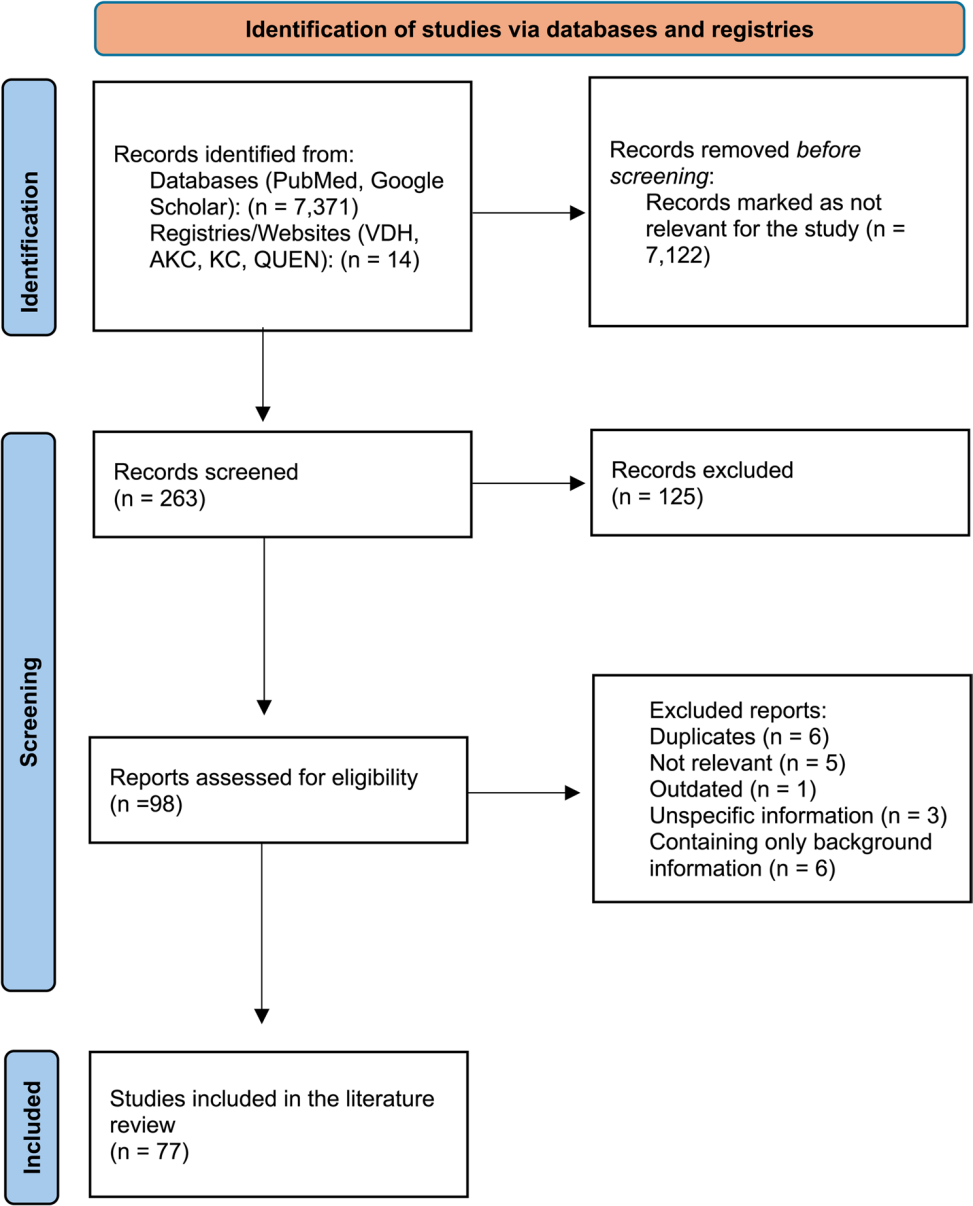
Literature review. This flow chart outlines the step-by-step process used to identify, screen, and include studies for the literature review in the context of the research

In April 2024, a comprehensive literature review was conducted, evaluating scientific publications (primary literature) focused on overall health, specific diseases, and current research trends related to the French bulldog breed (Fig. 1). Based on this comprehensive search, the most frequent disorders in the general dog population and, more specifically, the French bulldog were determined. This information served as the basis for developing the comprehensive study questionnaire aimed to be completed by French bulldog owners and including a series of structured questions evaluating the dogs’ health histories (Supplement 1). A second literature review was conducted in January 2025 to gather additional background information relevant to the results. Primary databases used for this review included PubMed and Google Scholar, along with official sources such as the American Kennel Club (AKC), the UK Kennel Club websites, and the QUEN (*Qualzucht-Evidenz-Netzwerk*) database, to evaluate current breeding standards and registration data. Keywords including *French bulldog*,* brachycephalic*,* purebred*,* pedigree*,* Robinow-like syndrome*,* BOAS*, and *IVDD* guided the search, allowing for the identification of 77 relevant papers and sources. The majority of these publications (2004–2024) included either case reports on specific diseases affecting French bulldogs (*n* = 13) or case-control studies comparing French bulldogs with other breeds (*n* = 28). Studies that informed about general health issues, pathologies, and the physiology of the development of certain diseases in French bulldogs and the general dog population were reviewed to develop a deeper understanding of breed-related conditions (*n* = 36).

### Design of the study survey

This survey received ethical approval from the ethics committee at the Leipzig University Faculty of Veterinary Medicine (reference number EK 10/2025). All participating dog owners were fully informed about the purpose and scope of the study and provided their voluntary, informed consent to participate. The survey was organized into several subsections to simplify and structure its completion for participating owners, and it incorporated different types of questions. Depending on the information to be acquired, these questions were framed in a single-choice (e.g., sex, neuter status), multiple-choice (e.g., feeding habits), or matrix format (e.g., age at diagnosis of a certain condition). Furthermore, owners were given the opportunity to add additional information or further specification in a free-text format (open-ended questions). Mandatory information pertained to age, death, weight, purebred status, and origin of the dog.

The survey was available in German and English between April and June of 2024 (9 weeks) and was promoted online via social media channels, including 56 different Facebook groups. Participating owners were informed about the purpose of the survey-based study, as well as the general requirements and conditions. These included the requirement that a veterinarian must have previously diagnosed any disease or condition entered in the survey. Only data extracted from completed and actively transmitted surveys were used for the data analysis.

### General characteristics

**Fig. 2:**
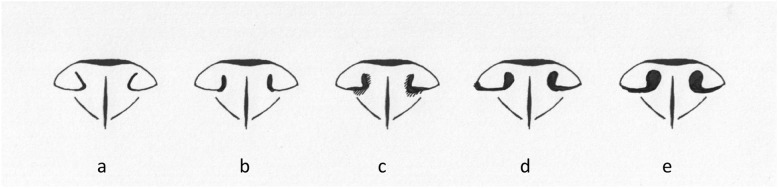
Assessment of the nostrils. This image panel shows the possible grades for the opening of the nostrils in French bulldogs observed by their owners, from narrow (grade 1; left image) to wide (grade 5; right image). Drawings by Anne Dohrmann. **a** Nostrils nearly closed, with hardly any visible opening seen between the lateral and median aspects of the nostril. **b **Nostril that is mostly closed, but with a small opening between the lateral and median aspects of the nostril. **c **Narrow nostrils where the lateral aspect touches the median part at the upper portion of the nostril, and the nostrils are only open at the lower portion. **d** Mildly narrow nostrils, where the lateral aspect does not touch the median part of the nostril. **e** Wide open nostrils (considered as normal)

**Fig. 3:**
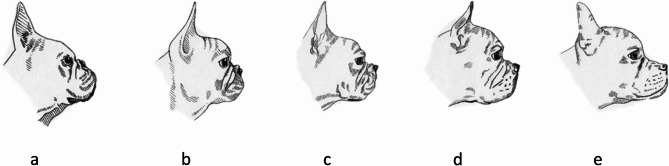
Assessment of the length of the nose. This image panel shows the possible lengths of the nose from French bulldogs observed by their owners, from nearly no protruding nose (left) to a close to mesocephalic shape of the head (right). Drawings by Anne Dohrmann.** a** Extremely short nose: The muzzle is almost absent, with the nasal plane positioned very close to the eyes, giving the face a markedly flattened appearance. **b** Severely shortened nose: The nasal length is minimal, and the face remains strongly brachycephalic, though the muzzle is slightly more distinct than in (a). **c** Moderately short nose: The muzzle is visible and distinct but still short relative to skull length. **d **Mildly short nose: The muzzle extends further forward, producing a more balanced facial profile while retaining brachycephalic features. **e **Normal or nose: The muzzle length is proportionate to the skull, resembling a more moderate or mesocephalic configuration

Owners were asked to respond to general questions about their dog’s general characteristics, including the dog’s name, age, sex, reproductive status, origin, color, body weight, body height, food and feeding practices, lifestyle, and pet health insurance status. For individual criteria or features, panels or charts with exemplary images were provided as a visual aid for an accurate selection of the specific response (e.g., coat color, measurement of the dog’s height). Different widths of the nostrils (Fig. 2) and lengths of the nose (Fig. 3) were also illustrated, allowing owners to select their responses after comparing their dogs’ features to the graded characteristics in the image panels.

### Definitions

The description of BOAS as commonly used in the scientific context, which was also applied in this study, includes stenotic nares (Fig. 2a, b and c), an elongated soft palate, dyspnea, permanently increased respiratory sounds, and heat stress [30]. The owners’ response to the question whether their dog has or had BOAS had to be based on a diagnosis documented by a veterinarian.

### Disorders categorized by organ system

One hundred nine of the most common conditions in the general dog population and/or the French bulldog breed, determined based on the literature review, were allocated within the survey based on the primary organ system involved (Supplement 1). Owners were guided through the different subsections of the survey, organized by affected organ systems (e.g., gastrointestinal tract, skin, eyes, respiratory tract). With each subsection, the owners were asked to select whether and if, at what age, the dog was diagnosed with a possible disease or affected by a possible condition. For selected disorders (e.g., food hypersensitivity or allergy, intervertebral disc disease [IVDD], urolithiasis), additional questions were included for further characterization, and the option for free-text responses was provided to identify yet unknown diseases or conditions in this breed. In addition, at the conclusion of each subsection, owners could enter further information about their dog (e.g., diseases not listed).

### Behavioral characteristics

The survey further included questions to determine the general attitude and behavioral characteristics of the dogs in specific (e.g., confronting or high-stress) situations, such as during interactions with other dogs or loud noises. The owners had to classify their dogs’ behavioral patterns (aggression, fear, joy, indifference) in 8 different daily situations/interactions (interaction with known/unknown dogs, interaction with known/unknown humans, interaction with kids, interaction with men, loud noises, being alone).

### Data Preparation and cleaning

**Fig. 4:**
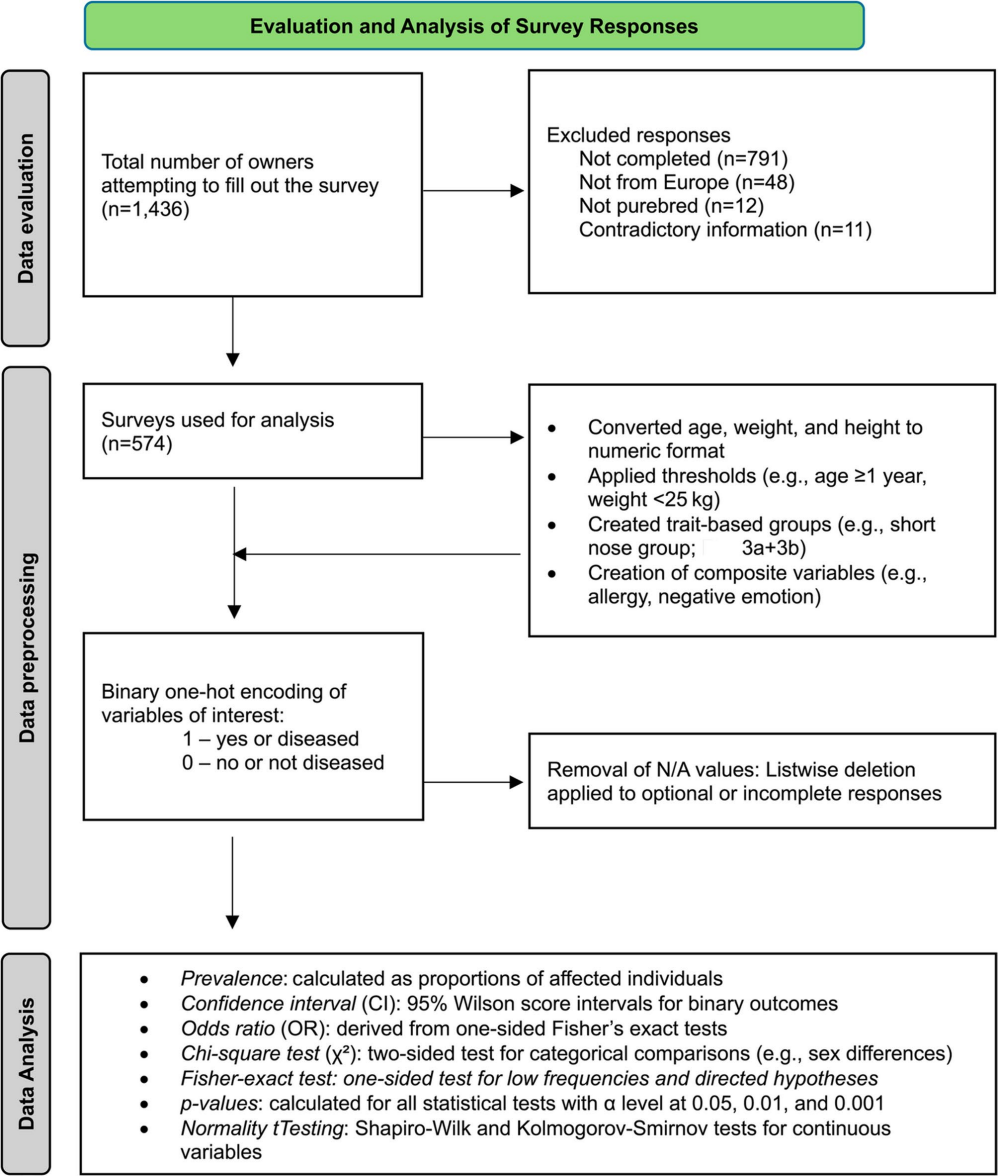
Process of the data analysis from data evaluation to processing and analysis. This flow chart outlines the systematic process of evaluating, preparing, and analyzing survey data collected from dog owners. The chart illustrates the sequential steps of filtering of valid responses, preprocessing of survey variables, group formation based on predefined criteria, and the statistical methods applied during analysis

The dataset was initially organized and preprocessed in Microsoft Excel (Version 16.88, Microsoft Corporation, Redmond, WA, USA). Surveys with incomplete responses, contradictory information, or dogs not meeting the criteria for purebred French Bulldogs were excluded. Column names were manually renamed to facilitate data processing (Fig. 4). Optional questions (e.g., tail length, nose shape, behavioral traits) were handled via listwise deletion, and analyses involving those variables were conducted on the reduced subset of valid responses to avoid biasing prevalence estimates. Missing values were systematically removed during preprocessing. Further data cleaning and transformation steps were conducted using Python (Version 3.12.8), *pandas*, and *numpy*. One-hot encoding was applied to binary illness indicators. Variables such as age, weight, and height were converted to numeric format with appropriate filtering thresholds (e.g., age ≥ 1 year, weight < 25 kg). All ‘yes/no’ responses were mapped to a binary format (1/0). New composite variables were generated for analyses, such as *negativ_emotion*, which captures undesirable behavior when a dog was reported to have exhibited aggression or fear at least once. The French bulldogs included in this study were further stratified based on nasal characteristics using visual references. Dogs with nostrils corresponding to reference images in Fig. 2a-c were classified as having stenotic nares, whereas 2 d was considered mild stenosis and 2e as non-stenotic (modified from Liu et al. 2016, *The mobility of the brachycephalic canine nostril in relation to the degree of nostril stenosis*, and Ravn-Mølby et al. 2019, *Breeding French bulldogs so that they breathe well—A long way to go).* Nose lengths were grouped using images in Fig. 3a-e: 3a + b indicated short noses; Fig. 3d + e and those with comparatively longer noses, not shown in any of the figures; and dogs matching Fig. 2c were excluded from relevant analyses.

### Statistical analysis

As an initial exploratory step, descriptive analyses were performed to examine the distribution of continuous variables, including the dogs’ age, weight, and height. Further analyses were conducted using Jupyter Notebooks within Microsoft Visual Studio Code (VS Code) as the development environment. Python and its scientific libraries (*pandas*, *scipy*, *statsmodels*, *seaborn*, and *matplotlib*) were used for descriptive and inferential statistics. Continuous variables (*age*, *weight*, *height*) were assessed for normality using Shapiro-Wilk and Kolmogorov-Smirnov tests. Histograms with KDE (Kernel Density Estimate) plots and q-q plots were generated to visually support normality assessments. Comparative statistics between male and female dogs were performed using a non-parametric Mann-Whitney *U* test for continuous variables. For categorical variables, differences in the prevalence of illnesses or traits between male vs. female dogs were assessed using a two-sided (undirected) Chi-square test. A one-sided Fisher’s exact test was used for directed hypothesis testing, specifically to test whether a particular group (e.g., dogs with a specific trait or exposure) exhibited a higher risk of certain diseases or clinical signs. A Fisher’s exact test was applied when a directional relationship was suspected and when expected frequencies were low. This test was applied in an exploratory fashion across a wide range of variable combinations, particularly when a directional relationship was suspected. Both Chi-square and Fisher’s exact tests were performed using the *scipy.stats* module in Python. Significance levels were reported as *p* <.05: statistically significant (*), *p* <.01: highly significant (**), and *p* <.001: very highly significant (***). The same notations are used to indicate significance levels in Fig. 4.

Prevalence values were calculated as proportions, and Wilson confidence intervals (95% confidence level, α = 0.05) were computed to provide robust estimates for binary outcomes. Graphs and visualizations were generated using Seaborn and Matplotlib. Median survival time following tumor diagnosis was calculated in Microsoft Excel based on the difference between age at diagnosis and age at death.

## Results

Of 1,436 owners who started completing the survey, 574 completed and submitted the survey, making the data available for this study. Thus, data from 574 French bulldogs were extracted and analyzed, of which 499 dogs (87%) were still alive at the time of the survey, whereas 75 dogs (13%) were deceased.

### General characteristics

**Fig. 5:**
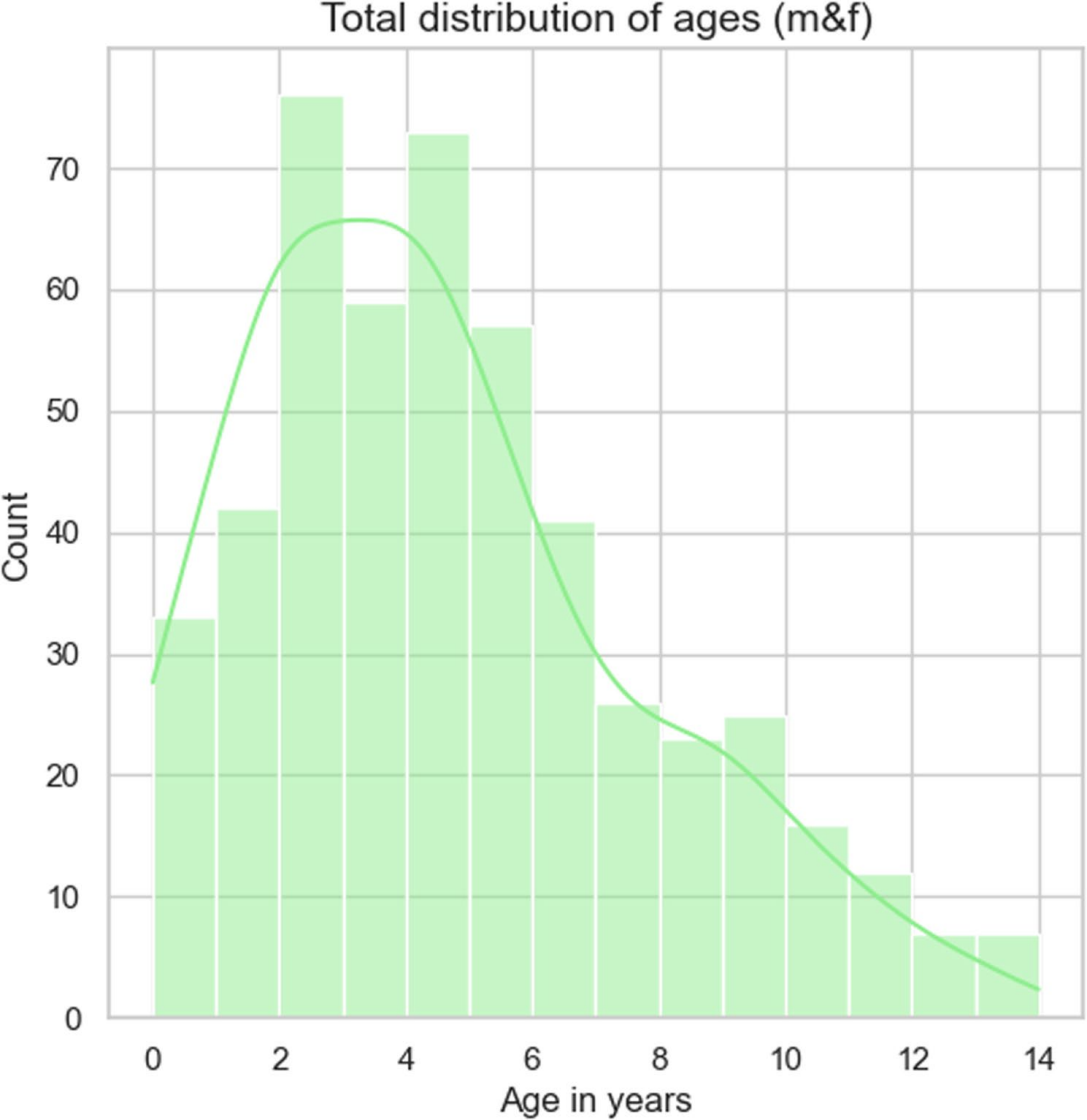
Age distribution of the dogs (alive at the time of the survey) included in the study. This figure illustrates the age distribution of all dogs included in the final analysis, combining both male and female individuals (*n* = 574). Age is displayed in full years on the x-axis, ranging from 0 to 14 years, while the y-axis indicates the count (frequency) of dogs within each age group. The green line represents the smoothed distribution curve

The female-to-male ratio was nearly even (Table 1), and about half of the dogs were intact (i.e., not spayed or neutered). The median age of the dogs alive at the time of the survey was 4 years (Fig. 5). Considering the adult body weight and size at ≥ 1 year of age, female dogs generally weighted less (median 12.1 kg; *p <*.001) and were smaller (median: 33.5 cm; *p* <.001) than male dogs (median weight: 14.2 kg; median height: 36.2 cm) (Supplement 1–4). The most common coat color included brindle (*n* = 136; 24%), followed by blue (*n* = 90; 16%). One hundred dogs (17%) could be phenotypically assigned to more than one coat color or pattern (Table 1). Dogs included in the study were born in one of 17 different European countries, with the largest proportion of dogs born in Germany (*n* = 405; 71%), followed by dogs originating from Hungary (*n* = 44; 8%). About half of the dogs (*n* = 285) were obtained from a breeder. More than half of the dogs were on a pet health insurance plan (*n* = 313; 55%), 180 of which had full insurance coverage for veterinary healthcare costs, and 133 had coverage for surgical procedures only (Table 1). One-fourth of all dogs received long-term medication (25%). 44 French bulldogs were used as breeding dogs.

**Table 1:** General characteristics of the French bulldogs (*n* = 574) included in the study. This table summarizes the sex, age (only dogs alive at the time of the survey), color, origin, and health insurance coverage of participating dogs

Variable	Category	Count (%)
Sex	Female	282 (49.1)
	Male	292 (50.9)
Neutered (surgically or medically)	Female	154 (55.0)
	Male	161 (55.1)
Age	*<* 1 year	33 (6.6)
	1–2 years	118 (23.7)
	3–4 years	132 (26.6)
	5–6 years	98 (19.7)
	7–8 years	49 (9.9)
	9–10 years	41 (8.2)
	11–12 years	19 (3.8)
	13–14 years	7 (1.4)
Health insurance coverage	Fully	180 (31.3)
	Partly	133 (23.2)
	Non	247 (43.0)
Origin	Germany	405 (70.6)
	Hungary	44 (7.7)
	Switzerland	32 (5.6)
	Austria	23 (4.0)
	Other	70 (12.1)
Color	Brindle	119 (20.7)
	Mixed	100 (17.4)
	Blue	90 (15.7)
	Pied	58 (10.1)
	Fawn	57 (9.9)
	Merle	48 (8.4)

Of the 75 dogs that were no longer alive at the time of the survey, 35 (47%) dogs had either died or been humanely euthanized because of underlying neoplastic diseases. Thirteen different tumor types as a cause for death were identified. (Table 2). From 42 entries stating their dog’s age at the time of death or euthanasia, the median age at death was 8.3 years.

**Table 2:** Causes of death or reasons for the decision to elect humane euthanasia (*n* = 75)

Cause of death or reason for humane euthanasia	Category	Count (%)
Neoplastic disease	Brain	5 (10.6)
	Unknown	5 (10.6)
	Lymphoma	4 (8.5)
	Liver	4 (8.5)
	Spleen	3 (6.4)
	Heart	3 (6.4)
	Mast cell	2 (5.7)
	Mammary gland	2 (5.7)
	Other	7 (20)
Cardiovascular decompensation/collapse		6 (8.0)
Epilepsy		4 (5.3)
Anesthesia incident		4 (5.3)
Unknown		4 (5.3)
Infectious disease		3 (4.0)
Intervertebral disc disease (IVDD)		3 (4.0)

### Conditions and diseases stratified by organ systems

Of the 574 dogs for which the survey was completed, 540 (94%) were reported to have been diagnosed with at least one condition (tail malformations excluded), rendering 34 dogs (6%) completely healthy at the time of owner participation in the survey; 47% of those dogs were within their first year of life.

The most frequently reported abnormalities or diseases are listet in Table 3.

**Table 3:** Most common disorders in French bulldogs in this study (*n* = 574). Comparison of their prevalence in female vs. male dogs

Disease/condition	Occurrence/Fraction	Total Prevalence	Confidence Interval (CI)	Female Prevalence	Male Prevalence	*p*-value
Tail malformation	504/515^a^	0.978	0.96–0.99	0.972	0.984	0.487
Allergy/Hypersensitivity	298/574	0.519	0.48–0.56	0.485	0.551	0.136
Otitis	206/574	0.359	0.32–0.40	0.369	0.349	0.690
Stenotic nares	191/544^a^	0.351	0.31–0.39	0.310	0.393	0.055
Pseudopregnancy	93/282^b^	-	-	0.330	-	-
Dystocia	18/60^c^	-	-	0.300	-	-
Elongated soft palate	152/574	0.265	0.23–0.30	0.255	0.274	0.681
Hemivertebrae	141/574	0.246	0.21–0.28	0.227	0.264	0.355
Conjunctivitis	124/574	0.216	0.18–0.25	0.199	0.233	0.370
Underbite	120/574	0.209	0.18–0.24	0.238	0.182	0.121
*Giardia spp.* in fecal samples	117/574	0.204	0.17–0.24	0.188	0.219	0.409
Skin fold dermatitis	113/574	0.197	0.17–0.24	0.177	0.216	0.292
Dental calculus	111/574	0.193	0.16–0.23	0.192	0.195	0.994
Intervertebral disc disease	105/574	0.183	0.15–0.22	**0.145**	**0.219**	**0.029**
Anal sac disease	89/574	0.155	0.13–0.19	0.174	0.137	0.271
Corneal ulcer	87/574	0.152	0.12–0.18	0.138	0.164	0.450
Cystitis	81/574	0.141	0.12–0.17	**0.177**	**0.106**	**0.020**
BOAS*	77/574	0.134	0.11–0.16	0.110	0.158	0.121
Atopic dermatitis	71/574	0.124	0.10–0.15	0.117	0.130	0.726
Pyometra	33/282	-	-	0.117	-	-
Patellar luxation	61/574	0.106	0.08–0.13	0.106	0.106	1.000
Pancreatitis	58/574	0.101	0.08–0.13	0.099	0.103	1.000
Cryptorchidism	29/292^d^	-	-	-	0.099	-
Demodicosis	48/574	0.083	0.06–0.11	0.081	0.085	0.980
Spondylosis	44/574	0.076	0.06–0.10	0.078	0.075	1.000
Osteoarthritis	44/574	0.076	0.06–0.10	0.078	0.075	1.000
Cataract	37/574	0.064	0.05–0.09	0.060	0.068	0.817
Retained deciduous teeth	32/574	0.055	0.04–0.08	0.046	0.065	0.418

^a^Response was voluntarily; non-respondents were excluded

^b^Only female dogs included

^c^Only whelping dogs included

^d^Only male dogs included

*Brachycephalic obstructive airway syndrome

values in bold font indicate significant differences between male vs. female dogs maybe put this in the next column

### Gastrointestinal conditions

Of all 574 dogs, 239 (42%) had dental, gingival, or jaw abnormalities. Oral diseases were inflammatory in 111 dogs (19%). Underbite (*n* = 120) or retained deciduous teeth (*n* = 32) were malformations detected in 139 dogs (24%). Dogs with dental tartar or plaque (*n* = 111; 19%) had concurrent evidence of stomatitis in 28 (25%) cases (Supplement 5).

Altogether, 387 (67%) French bulldogs showed clinical signs of a gastrointestinal disease at least once in their life. Food allergy/hypersensitivity affected nearly every other dog included in the study (*n* = 281; 49%), and 72% of the dogs received this diagnosis within the first 2 years of their life. The main allergens presumed or identified were different types of grain (69%), chicken (51%), and beef (19%). French bulldogs diagnosed with a food allergy/hypersensitivity were more likely to have atopic dermatitis (odds ratio [OR] = 5.1; *p* <.001), narrowed ear canals (OR = 4.7; *p* <.001), environmental allergies (OR = 4.0; *p* <.001), skin fold dermatitis (OR = 3.7; *p* <.001), pancreatitis (OR = 3.3; *p* <.001), otitis externa or media (OR = 2.8; *p* <.001), episodes of inflammatory anal gland disease (OR = 2.3; *p* <.001), and conjunctivitis (OR = 2.2; *p* <.001) (Supplement 6). The main clinical signs that led to the suspicion or diagnosis of a food allergy/hypersensitivity were licking of the paws (*n* = 199; 71%), pruritus (*n* = 188; 67%), and recurring diarrhea (*n* = 153; 54%). In 227 (81%) cases, the reduction of clinical signs was possible due to changes in food ingredients or the transition to a hypoallergenic therapeutic diet. In 68 dogs, medical intervention was needed for control of the allergy-associated clinical signs.

Pancreatitis was reported to affect 58 French bulldogs (10%) with at least one episode. Of these, 43 (74%) dogs had a prior diagnosis of food allergy or hypersensitivity. Pancreatitis was more frequently reported for neutered dogs (OR = 2.8; *P <*.001), with 44 neutered dogs compared to 14 intact ones.

Less frequently reported conditions were pyloric stenosis (*n* = 7), megaesophagus (*n* = 4), ulcerative colitis (*n* = 3), and diaphragmatic hernias (*n* = 2).

### Skin and ear disorders

Overall, 54% of the dogs exhibited some form of skin or hair abnormality (Supplement 7). The most frequent abnormality of the skin represented alopecia. 119 (21%) French bulldogs experienced alopecia at least once, with the following causes identified: allergies/hypersensitivity (*n* = 79), Demodex infections (*n* = 26), and color-mutant alopecia (*n* = 9). In 24 dogs (16 females and 10 males), the cause of alopecia remained unidentified.

Skin fold dermatitis emerged as the most common inflammatory skin disease, affecting 20% of the dogs (*n* = 113). Notably, in one-third of the cases (*n* = 33), the diagnosis was established in dogs younger than one year. Recurring or persistent episodes of clinical presentations were reported for 21 dogs, and an associated with food hypersensitivities/allergies was documented in 82 dogs (73%).

French bulldogs with a blue coat color (*n* = 90) more likely developed color-mutant alopecia (OR = 2.0; *p* =.196), pyoderma (OR = 1.8; *p* =.295), and chin pyoderma (OR = 1.6; *p* =.232) than dogs with other coat colors, but had lower odds of developing atopic dermatitis (OR = 0.8; *p* =.819), environmental allergies (OR = 0.6; *p* =.918), and hair loss (OR = 0.5; *p* <.992). However, not all of these associations reached statistical significance. Color mutant alopecia was also identified in dogs with other coat colors, including seven pied, two fawns, one black, and one dark brown French bulldog.

Pruritus and paw licking were among the most common skin signs of allergies. Of the 318 dogs (55%) that exhibited at least one episode of paw licking, allergies were identified as the underlying cause in 217 dogs (68%), while pruritus related to allergies was observed in 158 dogs (28%).

Otic conditions were reported in 39% (*n* = 221) of all dogs, including otitis externa (*n* = 165, 29%) and otitis media (*n* = 109, 19%). Among the dogs with otitis externa, 115 cases were associated with underlying allergies, 25 dogs developed an obstruction of the ear canal, and 34 dogs had recurrent otitis externa. Nine dogs experienced progressive deafness as a result of untreated or severe otitis.

### Respiratory diseases

**Fig. 6:**
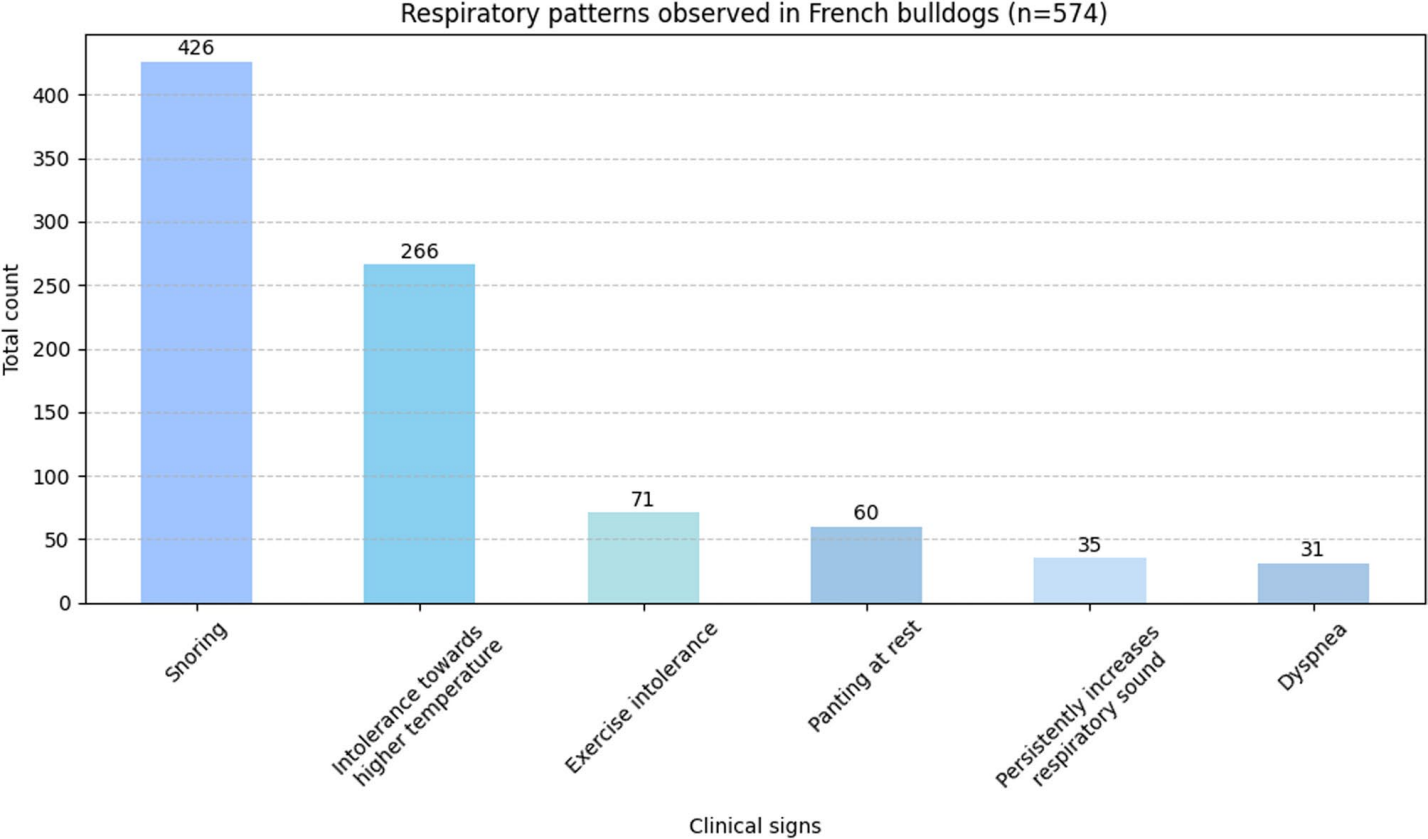
Respiratory patterns and limitations observed in French bulldogs (n=574). This bar chart visualizes the frequency of various respiratory-related clinical signs as reported in the population of 574 French bulldogs. The x-axis lists specific clinical signs, while the y-axis indicates the total number of dogs affected by each clinical sign.

Several different breathing patterns were described by the owners; 447 dogs (78%) snored while asleep, 281 dogs (49%) showed intolerance towards higher temperatures, and 75 dogs (13%) were classified as having exercise intolerance. Persistently increased respiratory sounds were reported for 62 dogs (10%). Among all French bulldogs included in the data analysis, 32 (6%) had experienced an episode of dyspnea at least once (Fig. 6).

Congenital, infectious, and degenerative conditions or abnormalities of the respiratory tract were documented in 283 dogs (49%) (Supplement 8). Congenital narrowing of the nostrils was the most common abnormality (*n* = 191; 33%) (Fig. 2a–c); 38 of these dogs (7%) had nostrils that were nearly closed, with hardly any visible space between the lateral and middle nostril wall (Fig. 2a). A slightly wider nostril that was still mostly closed, but with a small space between the lateral and medial (septal) aspect of the nostril was seen in 96 dogs (17%) (Fig. 2b). A nose where the lateral part of the nostril touches the medial aspect at the upper part of the nostril, and the nostrils are only open at the lower portion, was reported for 56 dogs (10%) (Fig. 2c). Nearly one-fifth of the dogs with stenotic nares (*n* = 37; 19%) were considered by their owners as having a normal unimpaired breathing pattern.

**Fig. 7:**
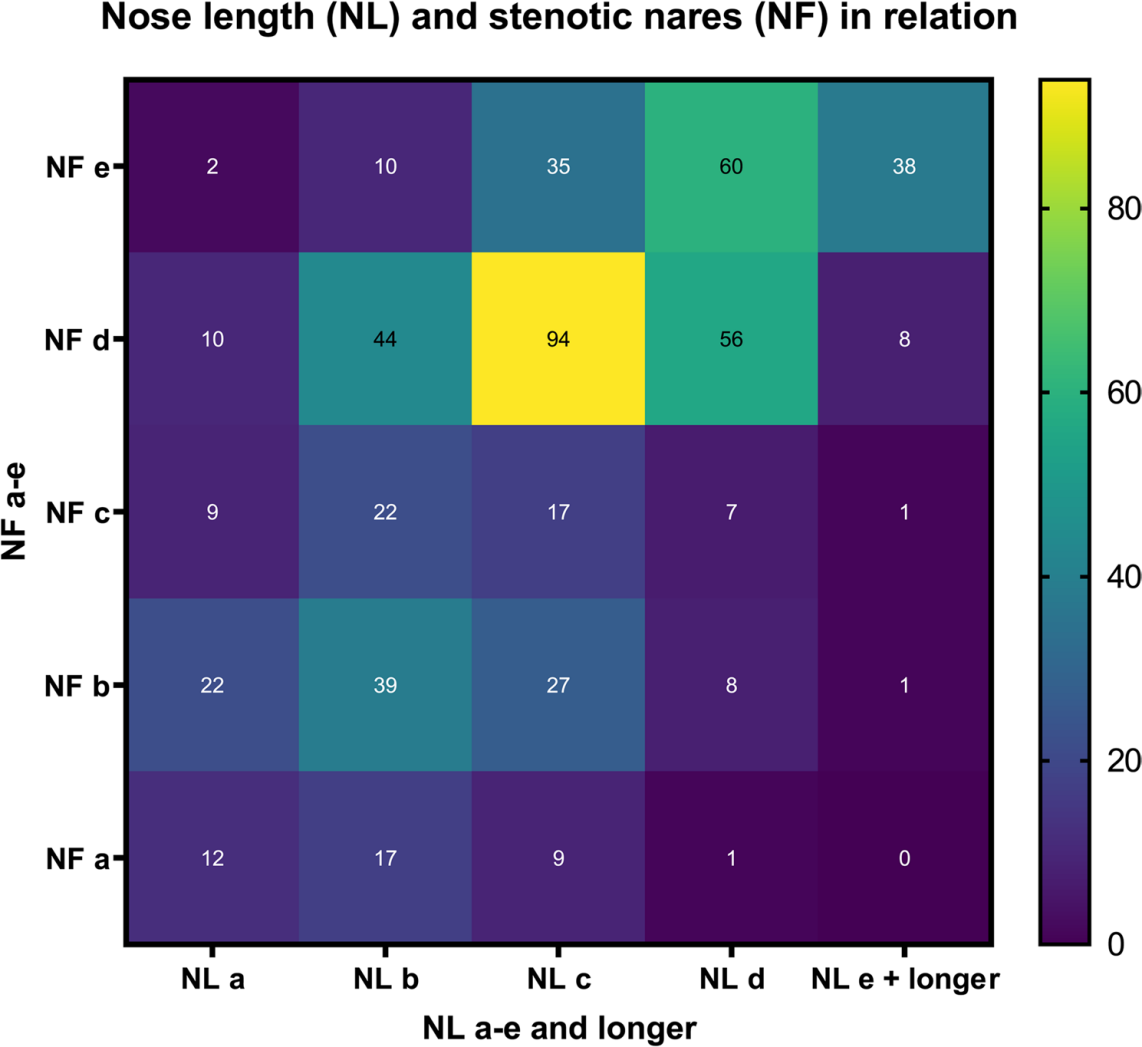
Heat map demonstrating the correlation between nose length (NL; x-axis) and the severity of stenotic nares (NF; y-axis). Nose length is categorized from a = very short to e = long, while stenotic nares range from a = nearly closed to e = fully open. Color intensity indicates the number of individuals per category combination, with brighter colors reflecting higher frequencies. This visualization highlights the distributional relationship between facial morphology and nostril conformation. For classification criteria, refer to Fig. 2 and Fig. 3

**Fig. 8:**
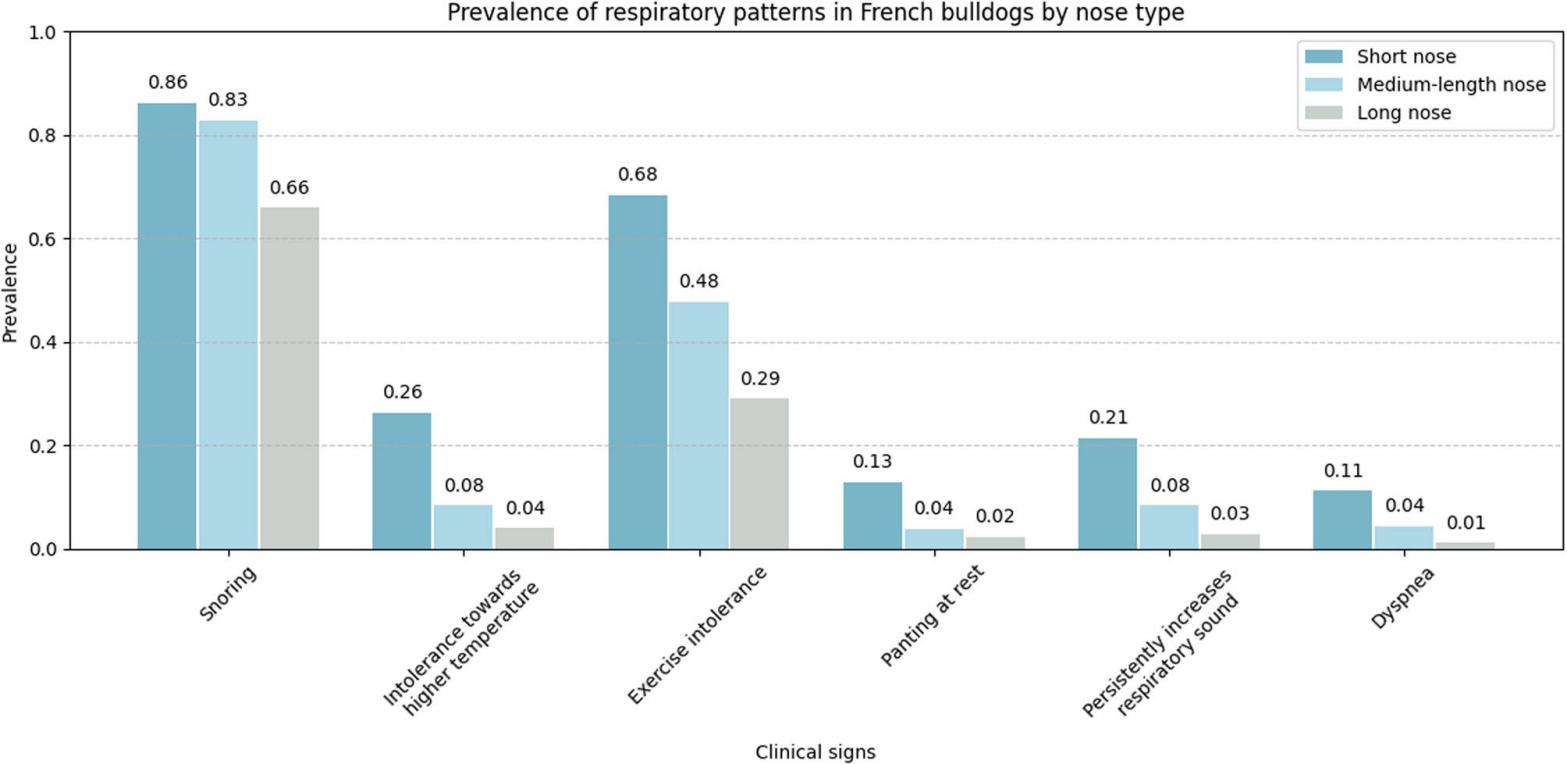
Respiratory patterns and limitations in the French bulldogs stratified by the length of the nose. Dogs were grouped based on nose length as follows: Short nose (see Fig. 2a + b). Medium-length nose (see Fig. 2c). Longer nose (see Fig. 2 d + e, and additional individuals with comparatively longer noses not depicted in the figures). The x-axis displays various owner-reported respiratory signs, including snoring, heat and exercise intolerance, panting at rest, persistent respiratory noise, and dyspnea. The y-axis represents the prevalence (proportion of affected individuals) within each nose-type group. Each bar indicates the relative frequency of the respective clinical sign in the three morphological subgroups.

French bulldogs with stenotic nares had shorter muzzles in comparison to French bulldogs without stenotic nares (OR = 7.9; *p* <.001)(Fig. 7). These dogs also had a higher prevalence of the conditions listed in Table 4; Fig. 8.

**Table 4:** Concurrent conditions of French bulldogs with stenotic nares (Fig. 2a and c) in this study (*n* = 191)

Disease/condition	Stenotic nares (*n* = 191)	Prevalence	Confidence Interval (CI)	Odds Ratio (OR)	*p*-value
Short muzzle	121	0.634	0.56–0.70	7.5	*<* 0.001***
Laryngeal collapse	10	0.052	0.03–0.09	6.4	0.002**
Persistent respiratory noise	43	0.225	0.17–0.29	5.7	*<* 0.001***
Exercise intolerance	50	0.262	0.20–0.33	5.6	*<* 0.001***
Hypoplastic trachea	19	0.099	0.06–0.15	5.5	*<* 0.001***
Panting at rest	25	0.131	0.09–0.19	5.2	*<* 0.001***
Snoring	175	0.916	0.87–0.95	4.4	*<* 0.001***
BOAS*	49	0.257	0.20–0.32	4.3	*<* 0.001***
Dyspnea	21	0.110	0.07–0.16	4.2	*<* 0.001***
Need for a second BOAS correction surgery	17	0.089	0.06–0.14	4.2	*<* 0.001***
Heat stress	132	0.691	0.62–0.75	3.7	*<* 0.001***
Elongated soft palate	83	0.435	0.37–0.51	3.6	*<* 0.001***

*Brachycephalic obstructive airway syndrome

*p <.05; ** *p* <.01; *** *p* <.001

A short skull with nearly no protuberant nose (Fig. 3) was reported for 57 French bulldogs (10%), and about a quarter of the dogs (*n* = 140; 24%) were reported to have a nose as shown in Fig. 3b. Most dogs were classified into the third category (*n* = 197; 34%) (Fig. 3c), 136 dogs (23%) had a longer nose (Fig. 3d), and 50 dogs (9%) a fairly normal nose (Fig. 3e). Dogs with shorter muzzles (Fig. 3a, b) were more likely to exhibit the conditions listed in Table 5.

**Table 5:** Concurrent conditions of French bulldogs with short noses (Fig. 3a and b) in this study (*n* = 198)

Disease/condition	Short nose (*n* = 198)	Prevalence		Confidence Interval (CI)	Odds Ratio (OR)	*p*-value	
Stenotic nares	121		0.647	0.58–0.71	7.5		*<* 0.001***
Hypoplastic trachea	20		0.101	0.07–0.15	5.9		*<* 0.001***
BOAS	52		0.263	0.21–0.33	5.0		*<* 0.001***
Dyspnea	22		0.111	0.07–0.16	4.6		*<* 0.001***
Elongated soft palate	91		0.460	0.39–0.53	4.4		*<* 0.001***
Persistent respiratory noise	41		0.207	0.16–0.27	4.4		*<* 0.001***
Exercise intolerance	49		0.247	0.19–0.31	4.4		*<* 0.001***
Panting at rest	24		0.121	0.08–0.17	4.2		*<* 0.001***
Need for one BOAS correction surgery	69		0.348	0.29–0.42	3.9		*<* 0.001***
Need for a second BOAS correction surgery	17		0.086	0.05–0.013	3.8		*<* 0.001***
Heat stress	135		0.682	0.61–0.74	3.4		*<* 0.001***
Laryngeal collapse	9		0.045	0.02–0.08	3.5		*<* 0.001***
Intervertebral disc disease	53		0.268	0.21–0.33	2.3		*<* 0.001***
Snoring	169		0.854	0.80–0.90	2.1		*<* 0.001***
Hemivertebrae	64		0.323	0.26–0.39	1.9		0.001**
Constricted ear canal	18		0.091	0.06–0.14	2.8		0.005**

*BOAS *Brachycephalic obstructive airway syndrome

*p <.05; **p <.01; ***p <.001

While 365 (64%) French bulldogs showed signs of BOAS as per definition (e.g., heat sensitivity, narrowed nostrils), only 77 (13%) were officially documented to be diagnosed with this condition by a veterinarian.

A quarter of all dogs (*n* = 152; 26%) had an elongated soft palate, which was the second most common congenital abnormality in the respiratory tract of the French bulldogs in this survey-based study.

**Fig. 9:**
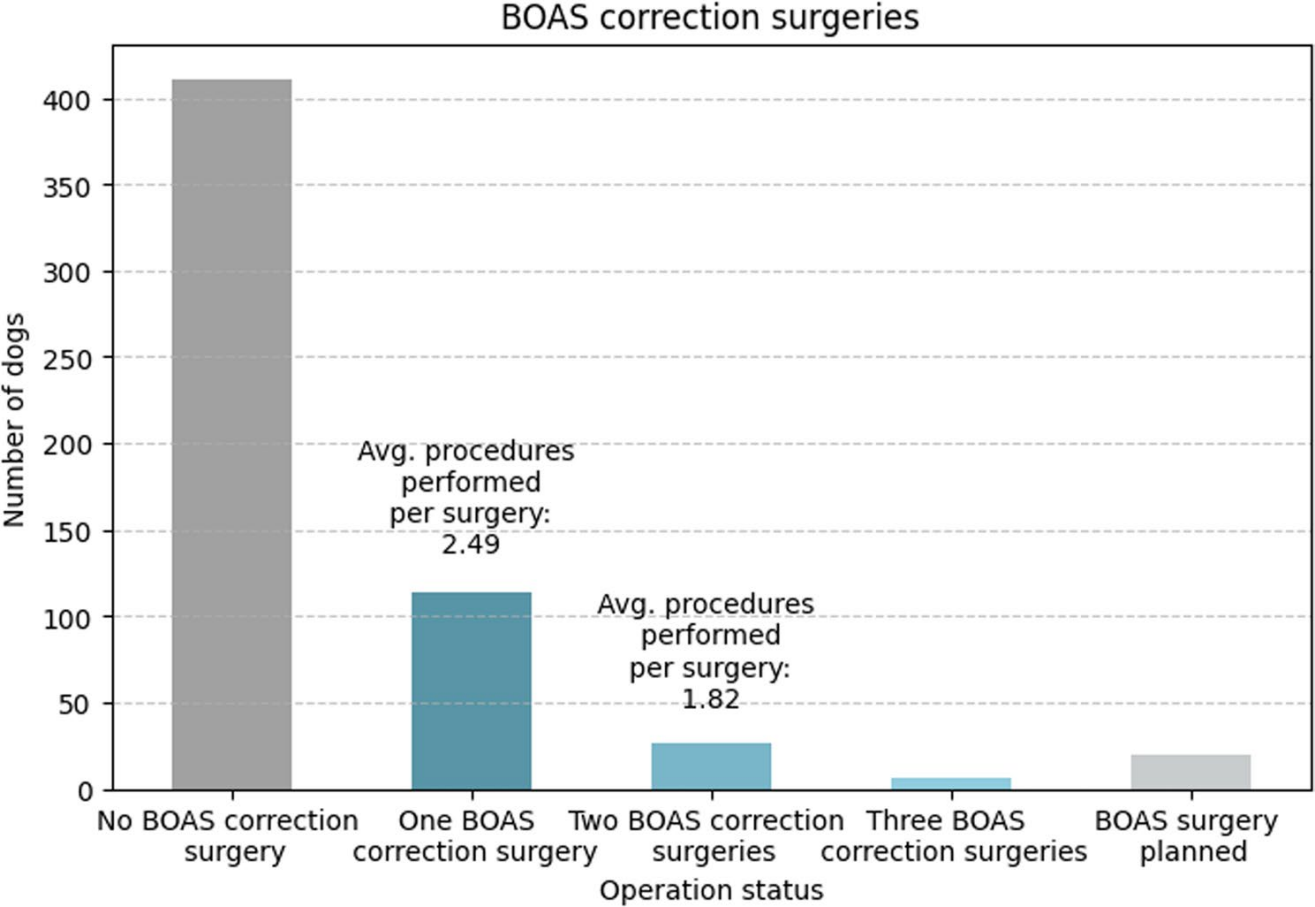
Frequency of BOAS (brachycephalic obstructive airway syndrome) correction surgeries among surveyed dogs, categorized by surgical status. The x-axis shows five categories: no BOAS correction surgery, one, two, or three BOAS correction surgeries, and BOAS surgery planned. The y-axis indicates the number of dogs per category. The calculation of average procedures per surgery was derived from owner-reported data, referring to interventions such as nostril widening, soft palate resection, or comparable BOAS-related corrections

More than one-fifth of the dogs (*n* = 134; 23%) had undergone or were scheduled to undergo at least one BOAS correction surgery (Fig. 9). The first brachycephalic correction surgery, performed in 114 (20%) French bulldogs, most frequently included shortening of the soft palate (*n* = 104; 91%) and widening of the nostrils (*n* = 96; 84%). In addition, one-fifth of the dogs had the tonsils removed or reduced (*n* = 24; 22%) and/or laser ablation of the nasal conchae performed (*n* = 24; 22%). In 26 dogs (23%), a second BOAS correction surgery was needed, where the nostrils were opened (*n* = 12; 46%), the soft palate shortened (*n* = 8; 31%), and the laryngeal pockets expanded (*n* = 7; 27%).

Less frequently reported conditions were tracheal collapse (*n* = 7), lungworm infections (*n* = 6), and chronic bronchitis (*n* = 4).

### Ophthalmologic disorders

Ocular diseases were diagnosed in 225 dogs (39%), with conjunctivitis being the most prevalent (*n* = 124; 22%), and over one-fifth of the dogs (*n* = 29; 23%) experienced more than one episode of conjunctivitis (Supplement 9).

Of the dogs diagnosed with conjunctivitis, 58% were ≤ 2 years old at the time of first diagnosis. French bulldogs with conjunctivitis were more likely to have skin fold dermatitis (OR = 3.6; *p* <.001), atopic dermatitis (OR = 3.4; *p* <.001), corneal ulcers (OR = 3; *p* <.001), and environmental or food allergies (OR = 2.5; *p* <.001) (Supplement 10).

Corneal ulcers were identified in 87 French bulldogs (15%), with 15 cases being attributed to a foreign body in the eye.

Among the dogs diagnosed with cataracts (*n* = 37), half had a history of corneal ulcers within two years of the diagnosis.

Additionally, 53 dogs (9%) were reported to have congenital eye abnormalities, including exophthalmos, entropion, and canine multifocal retinopathy (CMR).

### Musculoskeletal system diseases

213 (37%) French bulldogs had at least one degenerative, congenital, or traumatic disorder affecting the musculoskeletal system (congenital tail deformations not considered) (Supplement 11).

Within the population of 515 dogs evaluated for their tail length with this survey, 11 dogs (2%) had a long (i.e., normal) tail. Of the 504 dogs with a deformed or short tail, 12 had a malformed or cork-screwed tail, 155 (30%) showed no visible vertebral attachment, and more than half of the dogs (*n* = 338; 67%) had a short tail.

The most common abnormality in this subcategory was the presence of hemivertebrae, which were documented in 141 dogs, most of which were diagnosed between 1 and 2 years of age (*n* = 73, 52%). Of 71 owners knowing the subtype of hemivertebrae, 52 reported that wedge-shaped vertebrae were diagnosed in their dog. Dogs with hemivertebrae had a higher risk of developing osseous formations such as spondylosis (OR = 15.6; *p* <.001) or osteoarthrosis (OR = 5.8; *p* <.001) than dogs without hemivertebrae. Dogs with hemivertebrae also had a higher likelihood of concurrent disorders, including BOAS (OR = 4.2; *p* <.001), intervertebral disc disease (IVDD; OR = 4.2; *p* <.001), hip dysplasia (HD; OR = 3.9; *p* <.001), and patellar luxation (OR = 3.2; *p* <.001) (Supplement 12).

Patellar luxation was reported in 61 dogs (11%) with a median age of 3 years at the time of diagnosis. Osseus formations like spondylosis and/or osteoarthrosis were documented in 71 dogs (12%). Osseous formations were more common in neutered French bulldogs (*n* = 59; 83%) than in intact dogs (*n* = 12; OR = 4.7; *p* <.001).

Less frequent diagnoses included cruciate ligament rupture (*n* = 10), elbow dysplasia (*n* = 8), and primary inflammatory/rheumatic arthritis (*n* = 2).

### Female reproductive tract disorders

Data from 282 female dogs were included in this survey-based study, 155 (55%) of which were spayed, and 17 (6%) were spayed for health-related reasons such as pyometra or pseudopregnancy (Table 1).

Pseudopregnancy was the most common condition reported in all female dogs (*n* = 93; 33%), with 71 dogs (76%) showing typical behaviors or associated clinical signs within their first 2 years of life. Ten dogs had a second episode of pseudopregnancy with the following heat. Dogs with pseudopregnancy were more likely to develop pyometra (*n* = 17; OR = 6.5; *p* <.001) (Supplement 13).

Of the 60 female dogs that had at least one litter of puppies, 33 dogs were designated as breeding dogs. On a median, 5–6 puppies were born per litter, with a median of 2 litters over the lifetime of a breeding bitch. Complications during whelping were documented for 18/60 females (30%), of which 14 dogs (23%) underwent a C-section. In 9 of those dogs, it was due to a complication with puppies stuck within the birth canal (Supplement 14).

### Male reproductive tract disorders

Male dogs were less likely to be castrated than bitches (*n* = 137; 47%). Every tenth male dog was cryptorchid (*n* = 29), with the left or right testicle nearly equally affected (13:11; Supplement 13).

### Nervous system disorders

More than a fifth of all dogs (*n* = 126; 22%) were reported to have neurological diagnoses (Supplement 15). The most common neurological disorder was IVDD (*n* = 105; 18%), with more than half (61%) of the affected dogs diagnosed between 1 and 4 years of age. Most dogs had IVDD involving the lumbar spine (*n* = 74; 72%), followed by the cervical spine (*n* = 31; 30%). Return to normal function was reported for 71 dogs (67%), of which 23 (33%) had a second episode of IVDD elsewhere within the following two years. Neutered French bulldogs had higher odds for IVDD than intact dogs (OR = 2.4; *p* <.001), and male dogs had a higher risk of IVDD (*n* = 64) compared to females (*n* = 41; OR = 1.6; *p* =.014).

Epilepsy was reported for 21 French bulldogs (4%), first occurring at a median age of 6.5 years. In three dogs with epilepsy, a brain tumor was reported to have been diagnosed concurrently.

Less likely to appear were neuronal diverticula (*n* = 5), meningoencephalitis (*n* = 3), or fibrinoid leukodystrophy (*n* = 2).

### Urinary tract disorders

Urinary tract disorders were identified in 97 dogs (17%) in this survey-based study. The most common condition reported was cystitis (*n* = 81), with half of these cases occurring within the first two years of life. In 8 dogs, uroliths (struvite) were concurrently detected. Females were more frequently affected (*n* = 50) than males (*n* = 31; OR = 1.8; *p* =.010), and spayed females were more likely to develop cystitis than intact females (OR = 2.8; *p* =.002). No such difference was observed between neutered and intact males. Approximately 11% of dogs were reported to have recurrent cystitis episodes (Supplement 16).

Less frequently reported urinary tract conditions were chronic kidney disease (*n* = 4), renal dysplasia (*n* = 4), acute kidney diseases (*n* = 3), and leptospirosis (*n* = 1).

### Neoplastic diseases

Among the 574 dogs included in the study, 75 (13%) French bulldogs were diagnosed with at least one of 16 different neoplastic conditions (Supplement 17). Of these, 39 dogs succumbed to tumor-related effects or complications, while surgical intervention was successful in 30 cases. Mast cell tumors were the most common neoplastic condition, diagnosed in 26 dogs at a median age of 6 years (range: 1–12 years). These tumors were slightly more frequently observed in females (*n* = 16) than males (*n* = 10; OR = 1.7; *p* =.137) and were more prevalent in neutered dogs (*n* = 22) compared to intact dogs (*n* = 4; OR = 3.6; *p* =.005). The median survival time following diagnosis was 2–3 years.

Mammary gland tumors were the second most common neoplastic condition observed in French bulldogs in this study, diagnosed in 19 females at a mean age of 7 years (range: 3–12 years). Six dogs had previously whelped, and another six (OR 2.5; *p* =.070) had experienced at least one episode of pseudopregnancy. Among the affected dogs, seven underwent surgical tumor removal. The median survival time following diagnosis was 2–3 years. Splenic tumors were identified as the third most common tumor type (*n* = 11). The median survival time following diagnosis was 1–2 years. Three owners opted for surgical spleen removal, with two of these dogs surviving an additional 3.5–4.5 years post-surgery, while one remains alive. In contrast, dogs that did not undergo surgery had a significantly shorter lifespan of 0–1 year. Metastases or additional tumors were detected in seven cases around the time of diagnosis of the spleen tumor.

#### Cardiovascular disorders

Altogether, 37 French bulldogs (6%) were reported to have a cardiovascular condition (Supplement 18). These included arrhythmias (*n* = 9), pulmonary stenosis (*n* = 7), and endocardiosis (*n* = 6). A vector-borne systemic infection (*Babesia canis* [*n* = 2], *Leishmania infantum* [*n* = 3], *Dirofilaria immitis* [*n* = 3]) was diagnosed in 7 dogs. Coagulopathies were rare; only one dog was diagnosed with hemophilia.

### Endocrine disorders

Altogether 15 (3%) dogs were diagnosed with an endocrinopathy, the most common representing hypothyroidism (*n* = 8; 1%). Four of these dogs had concurrent pancreatitis at the time of diagnosis and/or an episode of pancreatitis earlier in life (OR = 9.5; *p* =.005). Less frequently reported were hyperadrenocorticism (*n* = 3), diabetes mellitus (*n* = 2), and hyperthyroidism of unknown etiology (*n* = 1).

#### Behavioral abnormalities

**Fig. 10:**
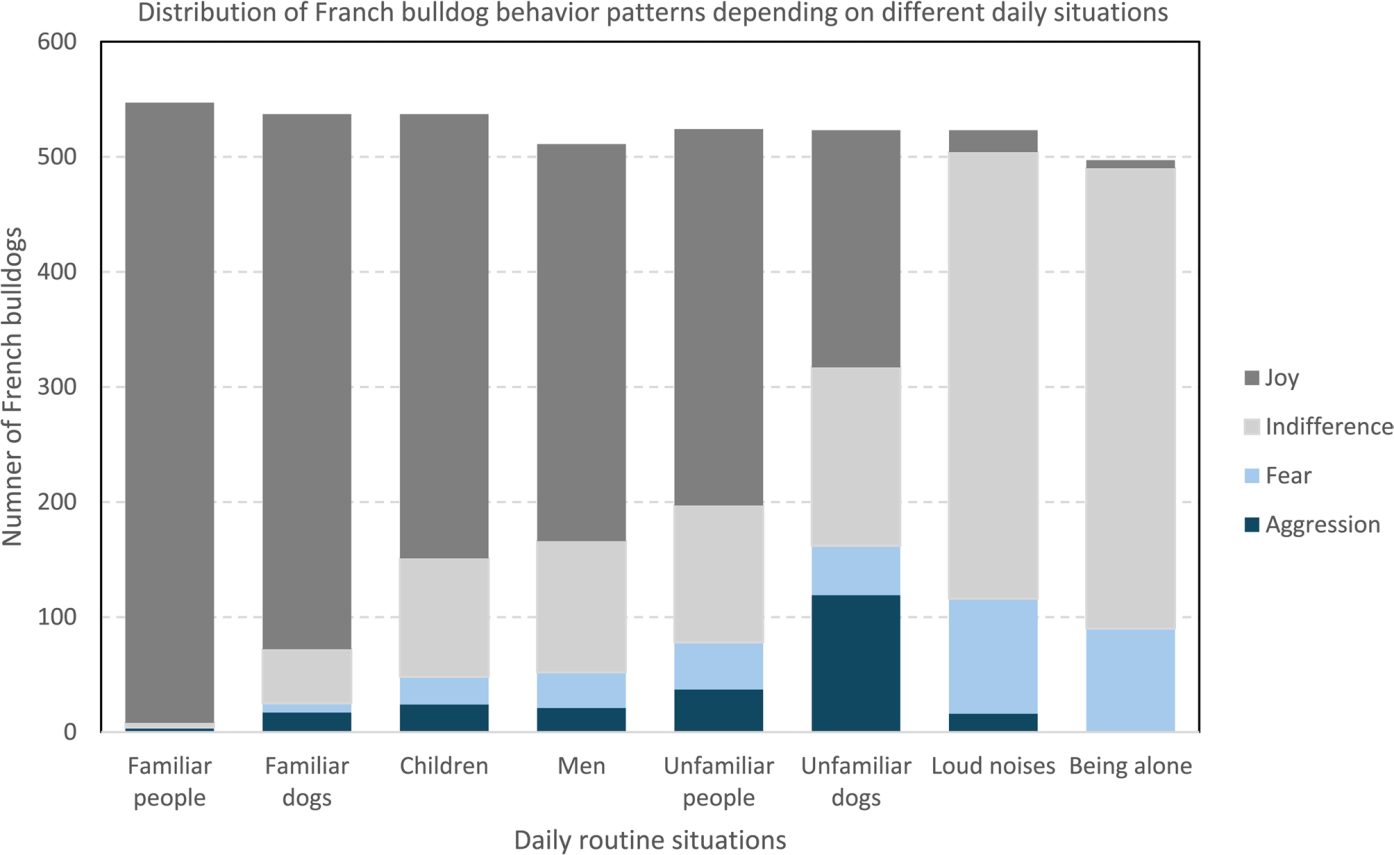
Distribution of behavioral and emotional patterns in different routine situations. This stacked bar chart shows the distribution of four behavior categories—joy, indifference, fear, and aggression—as reported by owners for different daily routine situations. The x-axis lists eight common scenarios (e.g., presence of familiar people, unfamiliar dogs, or being alone), while the y-axis indicates the number of dogs showing each behavioral pattern

**Fig. 11:**
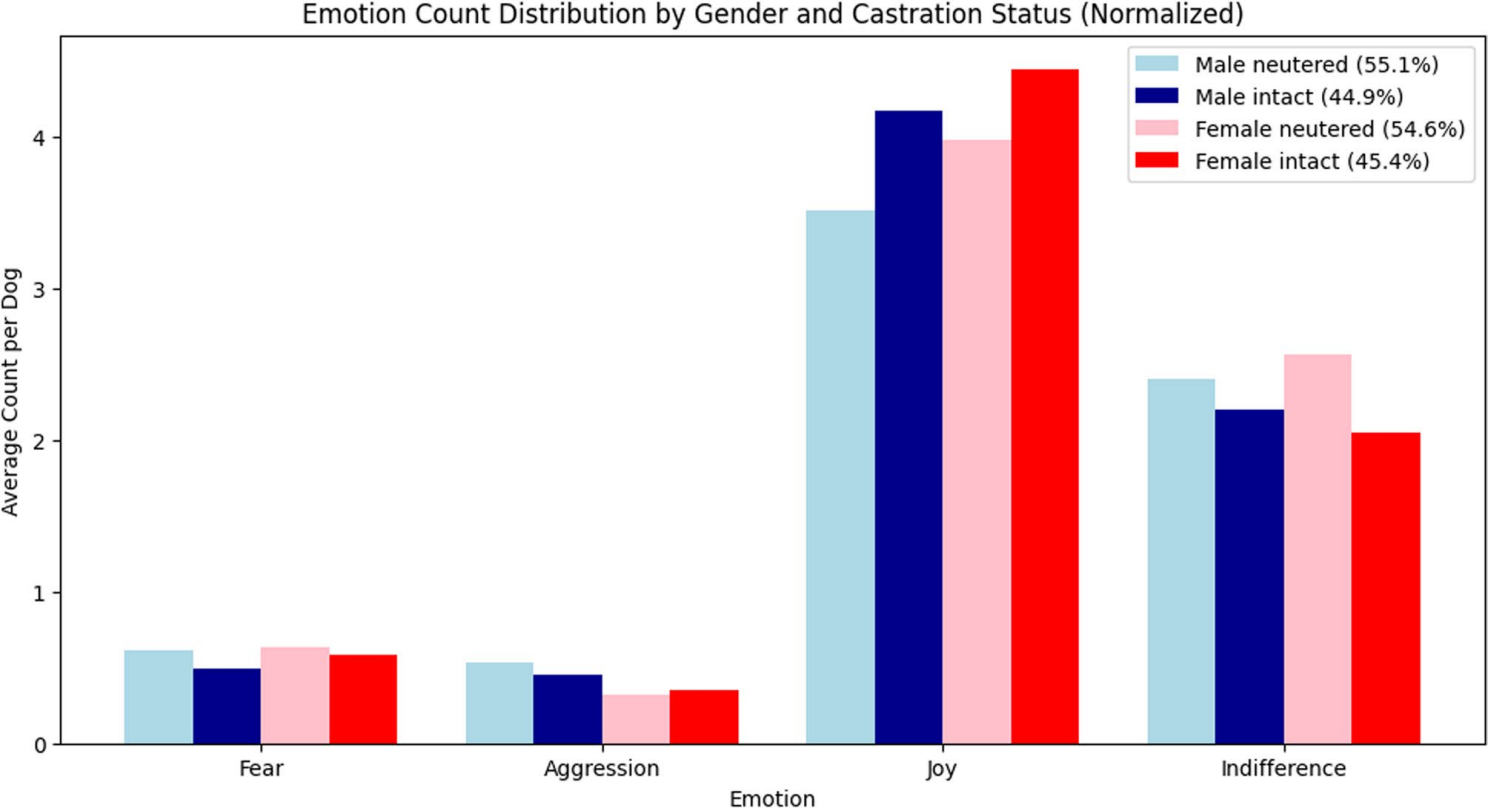
Distribution of selected behaviors and emotions stratified by sex and castration status. The chart displays the average number of situations (y-axis) in which each emotional response—fear, aggression, joy, and indifference (x-axis)—was observed, as reported by owners. A maximum of eight situations per dog was considered. Emotional patterns are stratified by sex and neuter status, represented by color-coded bars

In daily situations, most dogs exhibited either positive or neutral emotional responses. Joy was the most frequent interaction reported with familiar people (*n* = 539) and familiar dogs (*n* = 465). Conversely, loud noises (*n* = 19) and being left alone (*n* = 7) elicited the fewest joyful reactions (Fig. 10). In contrast, indifference was the predominant emotional response in situations where dogs were left alone (*n* = 400) or exposed to loud noises (*n* = 388). Aggressive behavior was relatively infrequent, with most instances directed toward unfamiliar dogs (*n* = 119) and, to a lesser extent, unfamiliar people (*n* = 37). Anxiety responses were most frequently observed in response to loud noises (*n* = 100) or being left alone (*n* = 89) and least frequently in interactions with familiar people (*n* = 0) and familiar dogs (*n* = 8) (Fig. 10 and Fig. 11).

## Discussion

This study aimed to explore the current overall and specific health status of French bulldogs in Germany based on a cohort of 574 dogs. The results of our survey-based study revealed that French bulldogs are prone to gastrointestinal, respiratory, and dermatologic conditions, which include various underlying etiologies and specific diseases. As a secondary aim of the study, we investigated whether French bulldogs displaying marked brachycephalic characteristics are more likely to incur other conditions.

Our data revealed a median life expectancy of 8.3 years for the French bulldogs included in the study. Compared to other dog breeds of the same size, this is rather young, as, for instance, the Beagle reaches an average lifespan of 10 years, and the Shih tzu has a distinctly higher life expectancy of 11 years [20]. The UK Kennel Club states that the lifespan of French bulldogs is over 10 years, while the AKC, the largest pedigree dog registry in the USA, estimates the average life expectancy of French bulldogs between 10 and 12 years [5, 31]. Our study suggests that French bulldogs in Germany lived for almost two years less than the AKC’s expectations. Interestingly, Reich et al. (2023) also reported a markedly shorter lifespan for French bulldogs at 7.7 years in Switzerland, whereas Teng et al. (2022) documented a life expectancy of 4.5 years and O’Neill et al. (2018) of 3.6 years for French bulldogs in the UK [20, 32, 33]. Inoue et al. (2018) report a median age of death for French bulldogs in Japan (10.2 years) that aligns with the AKC’s estimates [34]. These significant differences raise the question of whether French bulldogs in Japan are genuinely healthier than their European counterparts. However, a closer analysis suggests that the variations stem more from differences in study locations, methodologies, and data sources rather than actual health disparities. Compared to Teng et al., Inoue et al. reported substantially higher life expectancies — on average, five years more — across all breeds, not just French bulldogs [20, 34]. This discrepancy could be attributed to the data being based on crematorium records in Japan [34]. Pet cremation is widely practiced in Japan and is influenced by Buddhist traditions [35]. Cultural factors, such as significantly lower euthanasia rates, could contribute to a bias toward older ages [36, 37]. The result could be further skewed due to owners being more likely to elect cremation of a dog that lived longer rather than a dog that died young. However, Inoue et al. (2018) still place the French bulldog at the lowest end of life expectancies for all studied dogs, lending further support to the hypothesis that French bulldogs, in comparison to other breeds, live notably shorter [34]. O’Neill et al. studied a relatively young population of dogs with a median age of 1.3 years, potentially underrepresenting older dogs affected by disease or deceased due to natural causes [33]. The geographical proximity of Switzerland and Germany likely explains the similarity between the study by Reich et al. and our findings [32]. Discrepancies may also originate from differences in measurement approaches, such as median versus average life expectancy and whether stillbirths or early puppy mortality were included. These factors could significantly impact life expectancy estimates across studies.

As previously reported, the French bulldog breed was found to be affected by several health issues, including BOAS, otic diseases, presumed or confirmed allergies, and dystocia [4, 17]. Our study confirms several of these previous observations and further evaluates the French bulldog breed for less commonly known conditions. We found incidence rates for these diseases to be markedly higher than those previously reported from the UK (O’Neill et al. 2018) [33]. Some conditions (e.g., otitis externa) were 2.5 times more frequently reported, and others (e.g., patella luxation) occurred up to 5 times more often in our study than in the prior UK study [33]. However, despite these differences, both studies reach the same conclusions in view of the organ systems most commonly affected by disease, identifying the gastrointestinal tract and the skin to predominate [33]. For a breed that is primarily known for its respiratory problems, these results would support that other health issues may have been underreported or neglected, with attention centered on respiratory issues.

Surprisingly, food and environmental allergies or hypersensitivities were the most often reported condition, with an overall high prevalence (52%) in the French bulldog population included in this study. Clinical signs of an allergy can be evident on the skin and manifest as pruritus, causing generalized itching and other behaviors such as paw-licking [38]. Documenting such clinical signs in more than half of the dogs in this study was unexpected, given that others have reported significantly lower prevalences of allergies: Packer et al. (2019) found in their study that 28.9% of French bulldogs were affected by allergies, but O’Neill et al. (2021) reported only 3.2% of dogs with allergies [16, 17]. Picco et al. (2008) identified a prevalence rate of 2.3% for canine atopic dermatitis in French bulldogs, which may potentially be linked to allergies [39]. The differences between these studies may be linked to variations in the dog populations examined. For example, allergies might be underrepresented if the participating dogs are very young, as certain conditions may not have developed yet, as shown by O’Neill et al. (2021) reporting a median age of 1.5 years for French Bulldogs [17]. Regional variations in allergen exposure or differences in diagnostic procedures could also be important contributing factors [40]. With the rise in the popularity of French bulldogs in recent years, genetic bottleneck effects due to intense breeding could also contribute [8] as overbreeding can concentrate genetic predispositions to certain conditions (genetic bottleneck), including allergies, potentially leading to a higher prevalence in specific populations [41]. Differences in diet, lifestyle (e.g., outdoor vs. indoor time, use of allergen filters in air conditioning units), or environmental allergens (e.g., pollen, pollution, or chemicals) in the respective geographic area (Germany vs. the UK) could also contribute to the different allergy rates [40]. However, it is important to highlight that the 52% prevalence rate of allergies/hypersensitivity represents one of the highest reported in the current veterinary literature and may hold significant relevance for future research in this area. Particularly, the relation to secondary diseases such as otitis externa, skin fold dermatitis, and anal sac impactions, as well as their impact on the well-being of French bulldogs, should be carefully considered in future investigations [42].

This study further confirmed a predisposition of the French bulldog to reproductive issues. An expected finding was the high incidence of caesarean sections, with 23% of female dogs requiring surgical intervention during birthing due to anatomical constraints such as a narrow birth canal. Packer et al. (2019) reported an even higher rate of C-sections (31.6%), which is nearly 10% higher than in our study [16]. This difference may be attributed to variations in veterinary practices, including the growing preference for elective C-sections in the UK for breeds predisposed to dystocia, such as the French bulldog [43]. The high prevalence of pseudopregnancy (33%) in bitches observed in our study is a noteworthy finding. This figure may be even higher, given that 55% of the bitches in our study were spayed, some potentially before exhibiting signs of pseudopregnancy. Pseudopregnancy, or false pregnancy, is a physiological phenomenon typically accompanied by behavioral changes in female dogs at the end of metaoestrus [44]. Most bitches do not require veterinary intervention for this condition [45]. However, surgical intervention or medical treatment may become necessary if behavioral or physical changes, such as appetite loss, nesting behavior, aggression, or enlarged mammary glands, become severe and compromise the dog’s health or persist for an extended period [46]. Our results appear to be consistent with those for the general dog population, with Falceto et al. (2024) reporting an incidence rate of 30.8% for false pregnancy in dogs across all breeds in Spain [47]. Comparisons with other countries underscore challenges in data collection, including the higher neutering rates in the United States, which, in turn, places greater focus on neutered dogs [48]. A higher odds ratio (OR) was observed in our study in bitches experiencing pseudopregnancy in relation to mammary gland tumors, although the association was not statistically significant. Research on this correlation has been ongoing since the 1960 s, with earlier studies largely suggesting no significant association [49, 50]. More recent studies have documented a link between pseudopregnancy and the development of mammary gland tumors, likely due to hormonal influences [51–53]. These varying findings highlight the need for further research. Similar associations have also been observed between pyometra and pseudopregnancy, although the available data on this relationship remains limited [54–56].

The prevalence of congenital malformations in our study population was 65%, and presumably was largely due to selective breeding practices and breed standards. We excluded tail malformations from this analysis, as this feature is commonly present in the French bulldog population and would have skewed the results. According to the AKC, the breed’s tail is described as “straight or screwed (but not curly), short, carried low with a thick root and fine tip; held low when at rest”, which aligns with observing this breed standard in 98% of the dogs included in this survey-based study [5]. However, these tail malformations can be associated with Robinow-like syndrome, a genetic defect in the *DVL2* gene, which affects vertebral and cranial structures and is proposed to contribute to BOAS phenotypes [14, 57]. Mansour et al. (2018) and Niskanen et al. (2021) found alarmingly high prevalences (up to 100%) of homozygous *DVL2* deletion allele carriers (DVL2c.2044delC) in French bulldogs [14, 57]. Our data does not allow conclusions as to the proportion of French bulldogs potentially affected by Robinow-like syndrome, but vertebral malformations, such as wedge-shaped vertebrae, were reported for 23% of the French bulldogs in this study population. It can be reasonably assumed that the true prevalence of hemivertebrae is even higher, as not all dogs included in this survey-based study underwent screening diagnostic imaging to either confirm or exclude the possibility of this condition, particularly in asymptomatic dogs. In our study, French bulldogs with hemivertebrae were more prone to spinal issues, including IVDD, which has been linked to a *FGF4* mutation [57]. While genetic testing can help reduce the risk of breeding affected individuals, the *DVL2* mutation appears essentially fixed in French bulldogs [14]. This raises the question of whether crossbreeding may be the only viable long-term strategy to improve the overall health of the breed.

Robinow-like syndrome also affects the skull characteristics of French bulldogs, contributing to their most controversial feature: the extremely short nose [57]. Thus, this defect might be due to the selective breeding for extremely short noses (brachycephaly) over recent decades, an emphasized breed standard for French bulldogs [5, 11]. In our study, 35% of the dogs were reported to have an almost non-existent or very short nose. We found that these dogs were more susceptible to respiratory restrictions and airway malformations compared to those with longer noses. The skull characteristics lead to airway obstructions caused by an elongated soft palate, displaced nasal conchae, and narrowed nostrils, which force French bulldogs to compensate for reduced airway ventilation and oxygenation [11]. While some dogs are reported to maintain their oxygen supply via panting, other dogs exhibit early signs of respiratory limitations, such as exercise intolerance [58]. Interestingly, 32% of French bulldogs exhibited a longer nose, which can be regarded as a positive trait in modern breeding, as it may offer opportunities to select for improved respiratory conformation within the French bulldog breed.

In some cases of extreme malformations, respiratory distress can occur, creating a life-threatening situation [59]. These clinical signs, in combination, are summarized as BOAS [60]. Packer et al. (2015) proposed that 70% of French bulldogs suffer from BOAS, which aligns closely with our findings, suggesting that 64% of the French bulldogs included in the survey-based study are affected [12]. However, only 13% of the French bulldogs in our study had received a formal BOAS diagnosis from a veterinarian, highlighting two key issues. First, the challenges in diagnosing BOAS due to its broad and flexible definition; and second, the limited recognition of this condition, not only among owners but also among veterinary practitioners, with consideration of BOAS-related conditions such as panting and loud respiratory noises presenting “normal” breed-specific features. The substantial proportion of French bulldogs (22%) that underwent or were already scheduled for BOAS corrective surgery in our study is twice as high as described by Packer et al. (2019), underscoring the breed’s challenging anatomy and need for changes in breeding practices [16].

This survey-based study observed a low prevalence of cardiovascular diseases and endocrine disorders, aligning with the study by O’Neill et al. (2013), where cardiac and endocrine disorders had the lowest incidence rates [33].

Surprisingly, we found that the more commonly known diseases in French bulldogs were underrepresented, including the low incidence of histiocytic ulcerative colitis (granulomatous colitis) [61–63]. In our investigation, only three French bulldogs (0.5%) were affected by this condition, raising the question of whether the low numbers reflect the overall French bulldog population or challenges in diagnosing this condition play a role. Accurate diagnosis requires veterinary awareness and confirmation through biopsy sampling under general anesthesia, which can pose barriers to the owners’ compliance [64]. Although the breed is known to be susceptible, the true incidence remains undetermined in the literature. Furthermore, 62 owners (10%) reported that their dogs had persistently increased respiratory sounds — a relatively low figure given that 64% of French bulldogs in our study exhibited signs of BOAS. Considering that 34% had an extremely short nose (Fig. 3a and b), 33% had stenotic nares (Fig. 2a and c), 26% had an elongated soft palate, and 23% had undergone or were scheduled for airway correction surgery, the expectation would be a much higher prevalence of permanent respiratory noises. This discrepancy may be attributed to owners’ perception and gradual adaptation to their own dogs’ breathing habits, as well as the social normalization of such breathing impairments in the French bulldog breed [16]. In our study, 90 dogs with a single or mixed blue coat color participated. Several studies describe that blue-coated dogs, due to an autosomal recessive gene transmission, tend to develop color mutant alopecia, which manifests in a dry, poor hair coat and can progress to hair loss from the trunk to the flanks [65–67]. Our findings suggest a potential association between the blue coat color and color dilution alopecia; however, no such link was observed with other hair abnormalities or skin conditions, such as hair loss, atopic dermatitis, or allergies. It is important to note that these results were not statistically significant.

Section 11b of the German animal welfare law addresses the breeding of animals and prohibits the breeding of vertebrates if such actions are likely to cause hereditary physical or behavioral defects leading to pain, suffering, or harm in the animals or their offspring [68]. However, categorizing the degree of suffering in dogs proved to be difficult to assess because it is subjective and challenging without clear and objective criteria. The latest revision of Germany’s animal welfare law aims to address this issue by providing a more detailed definition of ‘dysfunction or malformation’, specifically targeting conditions like entropion, respiratory distress, or dental malformations [29]. However, the new legislation still leaves considerable room for interpretation. Under strict evaluation, this new policy could impact altogether 394 French bulldogs (69%) in our study, including 30 dogs that are used for breeding (68% of all breeding dogs), rendering only 31% of the dogs included in the survey suitable for breeding. Restricting breeding to 31% of French bulldogs could, in turn, significantly affect the genetic pool and, thus, the overall health of the breed, which already has limited genetic diversity. In 2016, the inbreeding coefficient for French bulldogs was estimated at 28%, and coefficients >10% signal an increased risk for the overall health of a breed, underscoring again the challenges involved in selective breeding practices [4]. A 2021 study at the University of California also found an inbreeding coefficient (F_adj_) of 22–28% for French bulldogs [69].

Notable shifts in the UK Kennel Club’s breeding statistics for French bulldogs have emerged. During the COVID-19 pandemic, new French bulldog registrations surged to a peak of 54,000 in 2021, only to drop by 51% within two years to just over 26,000 in 2023, reflecting a significant downward trend [8]. This decline aligns with a general decrease of 35% in registrations across all dog breeds [10]. However, the decline is particularly pronounced in brachycephalic breeds, such as the Pug (−67%), English bulldog (−55%), Boston terrier (−43%), and Boxer (−43%) [10]. One likely explanation is the reduced demand for pet dogs following the end of the COVID-19 pandemic. Additionally, increasing awareness of the health issues prevalent in these breeds — and the substantial costs associated with managing them — may have further contributed to this decline [16]. Beyond these practical considerations, social influences also must be considered. Ghirland et al. (2013) noted that owners often value fashion over functionality [70]. This raises the possibility that the French bulldog was subjected to a trend that is continued by other fashionable breeds, such as the long-haired Dachshund, which saw a 45% increase in registrations between 2021 and 2023 in the English KC statistics [10]. A similar decline in French bulldog registrations has not been observed in Germany (Lisa Frankenberger, personal communication, 19 September 2023). However, educating owners about breed-related health issues is likely a critical step toward improving the breed’s well-being and overall welfare concerns. This should generally take precedence over prioritizing appearance. Reaching out to and educating owners, however, remains the greatest challenge, requiring also effective collaboration between lawmakers and the veterinary profession.

Future investigations on French bulldogs could further explore areas to enhance understanding and improve the breed’s health and welfare. Comparison between French bulldogs and other dog breeds could also shed light on some unique health issues. Researching outcomes of crossbreeding with other dog breeds might present an option to reduce the frequency and severity of some hereditary conditions, as was shown to be successful in the cross-breeding of Dalmatians and Pointers [41]. Regular, standardized health checks for breeding animals could further help with the early detection of heritable disorders and slow-onset conditions over time, ensuring that primarily or only healthy dogs are entered into breeding programs [22]. Restrictions on breeding dogs with extreme anatomical features, as implemented in the Netherlands [28], could be expanded to implement the selection for anatomical and genetic diversity. This can reduce the prevalence of traits linked to severe health conditions and even behavioral characteristics and provide frameworks for shaping the future of breeding across dog breeds, ensuring healthier outcomes and improved welfare standards.

We acknowledge some limitations of this study. First, recruiting owners through Facebook allowed us to reach a broad and likely unbiased group, including both healthy and affected dogs. Although not a truly random sample, this recruitment strategy can be expected to provide a reasonably representative cross-section of the French bulldog population. Despite several refinements of the survey, the available question formats remain less than ideal and limit the specificity of some responses. The survey was not formally validated; however, it was tested for clarity and usability among different groups of individuals with and without veterinary or medical backgrounds. This shortcoming was particularly evident in the analysis for the age of the dogs at the time of neutering, categorization of clinical signs of abnormal breathing and nostril stenosis into pre- *vs.* post-BOAS corrective surgery, specification of disease resolution as either cured or having reached a subclinical state of the condition, precise age rather than assignment into age groups at 2-year intervals, and classification of repeated entries for episodes of the same disease presentation as either recurrence after full recovery or persistence of clinical signs. Second, the survey relied heavily on the pet owners’ understanding and awareness of various diseases. Although lay-worded explanations were provided to clarify certain conditions, some owners may have found the terminology of the survey challenging. Third, an owner’s knowledge of a condition affecting their dog is typically dependent on the condition being diagnosed or confirmed by a veterinarian. However, some diseases are difficult to diagnose or require an intensive diagnostic evaluation that owners may not consent to for financial or other reasons. That applies particularly to some conditions, such as hemivertebrae, which require radiographic or CT imaging for a definitive diagnosis and may be asymptomatic or produce slow-onset clinical signs in dogs, leading to a potential underdiagnosis. Thus, the true prevalence of such conditions could be significantly higher than that resulting from this survey-based study. Finally, due to the large size of this dataset, some associations may go unrecognized in the data analysis. Future reanalysis of the data could, therefore, shed light on new and unexplored associations. Despite these limitations, the responses from this survey provide valuable data that reveal important aspects of the current health status of the French bulldog in Germany and reflect areas that hold potential for significantly improving the health of the breed. 

## Conclusion

This survey-based study provides a comprehensive overview of the health status of French bulldogs in Germany. Allergies, congenital malformations, breathing restrictions, and skin problems emerged as the most prevalent issues. Furthermore, correlations were identified between nose length, breathing difficulties, and overall health, between allergies and concurrent conditions (e.g., anal sac impactions, conjunctivitis), and between congenital malformations and related complications (e.g., hemivertebra, IVDD, BOAS, and other respiratory problems). This study updates the general health status of French bulldogs in Germany and is therefore important to reduce disease prevalence and improve the long-term health and welfare of the breed.

## Supplementary Information

Supplementary Material 1.

Supplementary Material 2.

Supplementary Material 3.

## Data Availability

Anonymized data are available by the authors upon reasonable request..

## References

[1] Coile DC, Earle-Bridges M. French bulldogs : everything about purchase, care, nutrition, behavior, and training. Hauppauge, NY : Barron’s. 2005. p.102. Available from: http://archive.org/details/frenchbulldogsev00coil.

[2] Markovics J. French Bulldog: The Frenchie. Bearport Publishing. 2010:36.

[3] Verband für das Deutsche Hundewesen (VDH). Französische Bulldogge - Die offizielle Website für VDH-Welpen. Available from: https://welpen.vdh.de/hunderassen/rasselexikon/ergebnis/franzoesische-bulldogge. Accessed on date 2025 Feb 21.

[4] Grote K. Merkblatt Hund Rasse French Bulldog. QUEN Qualzucht-Database. 2023. Available from: https://qualzucht-datenbank.eu/merkblatt-hund-rasse-french-bulldog/. Accessed on date 2024 Dec 18.

[5] American Kennel Club. French Bulldog Dog Breed Information. Available from: https://www.akc.org/dog-breeds/french-bulldog/.

[6] TASSO e.V. Die beliebtesten Hunderassen. *TASSO Haustierregister.* Available from: https://www.tasso.net/Service/Wissensportal/TASSO-Fakten/Die-beliebtesten-Hunderassen. Accessed on date 2024 Dec 18.

[7] Loeb J, Williams A. French bulldogs still as popular as ever. Vet Rec. 2022;190(12):480. 10.1002/vetr.1906. 35714008 10.1002/vetr.1906

[8] The Kennel Club. 10-yearly breed statistics. Available from: https://www.thekennelclub.org.uk/media/tm3bsfgn/10-yearly-breeds-stats-utility.pdf. Accessed on date 2024 Dec 18.

[9] TASSO e.V. TASSO Haustierregister. Available from: https://www.tasso.net.

[10] The Kennel Club. Breed registration statistics. Available from: https://www.thekennelclub.org.uk/media-centre/breed-registration-statistics/.

[11] Ekenstedt KJ, Crosse KR, Risselada M. Canine brachycephaly: anatomy, pathology, genetics and welfare. J Comp Pathol. 2020;176:109–15. 10.1016/j.jcpa.2020.02.008. 32359622 10.1016/j.jcpa.2020.02.008 PMC7380493

[12] Packer RMA, Hendricks A, Tivers MS, Burn CC. Impact of facial conformation on canine health: brachycephalic obstructive airway syndrome. PLoS One. 2015;10(10):e0137496. 10.1371/journal.pone.0137496. 26509577 10.1371/journal.pone.0137496 PMC4624979

[13] Mitze S, Barrs VR, Beatty JA, Hobi S, Bęczkowski PM. Brachycephalic obstructive airway syndrome: much more than a surgical problem. Vet Q. 2022;42(1):213–23. 10.1080/01652176.2022.2145621. 36342210 10.1080/01652176.2022.2145621 PMC9673814

[14] Mansour TA, Lucot K, Konopelski SE, Dickinson PJ, Sturges BK, Vernau KL, et al. Whole genome variant association across 100 dogs identifies a frame shift mutation in DISHEVELLED 2 which contributes to Robinow-like syndrome in Bulldogs and related screw tail dog breeds. PLoS Genet. 2018;14(12):e1007850. 10.1371/journal.pgen.1007850. 30521570 10.1371/journal.pgen.1007850 PMC6303079

[15] Grote K. Merkblatt Hund Robinow-Like Syndrom. *QUEN* Qualzucht-Database. 2024. Available from: https://qualzucht-datenbank.eu/merkblatt-hund-robinow-like-syndrom/. Accessed on date: 2024 Dec 18.

[16] Packer RMA, O’Neill DG, Fletcher F, Farnworth MJ. Great expectations, inconvenient truths, and the paradoxes of the dog-owner relationship for owners of brachycephalic dogs. PLoS One. 2019;14(7):e0219918. 10.1371/journal.pone.0219918. 31323057 10.1371/journal.pone.0219918 PMC6641206

[17] O’Neill DG, Packer RMA, Francis P, Church DB, Brodbelt DC, Pegram C. French bulldogs differ to other dogs in the UK in propensity for many common disorders: a VetCompass study. Canine Med Genet. 2021;8(1):13. 10.1186/s40575-021-00112-3. 34911586 10.1186/s40575-021-00112-3 PMC8675495

[18] Töpfer T, Köhler C, Rösch S, Oechtering G. Brachycephaly in French bulldogs and pugs is associated with narrow ear canals. Vet Dermatol. 2022;33(3):214. 10.1111/vde.13067. 35293639 10.1111/vde.13067

[19] O’Neill DG, Rowe D, Brodbelt DC, Pegram C, Hendricks A. Ironing out the wrinkles and folds in the epidemiology of skin fold dermatitis in dog breeds in the UK. Sci Rep. 2022;12(1):10553. Available from: 10.1038/s41598-022-14483-5. 10.1038/s41598-022-14483-5 PMC9259571 35794173

[20] Teng KT-Y, Brodbelt DC, Pegram C, Church DB, O’Neill DG. Life tables of annual life expectancy and mortality for companion dogs in the United Kingdom. *Sci Rep.* 2022;12(1):6415. Available from: 10.1038/s41598-022-10341-6. 10.1038/s41598-022-10341-6 PMC9050668 35484374

[21] Aron DN, Crowe DT. Upper airway obstruction general principles and selected conditions in the dog and cat. Vet Clin North Am Small Anim Pract. 1985;15(5):891–917.10.1016/S0195-5616(85)50101-1. 2416110 10.1016/s0195-5616(85)50101-1

[22] Menor-Campos DJ. Ethical concerns about fashionable dog breeding. Animals. 2024;14(5):756. 10.3390/ani14050756. 38473141 10.3390/ani14050756 PMC10930939

[23] Sandøe P, Kondrup SV, Bennett PC, Forkman B, Meyer I, Proschowsky HF, et al. Why do people buy dogs with potential welfare problems related to extreme conformation and inherited disease? A representative study of Danish owners of four small dog breeds. PLoS One. 2017;12(2):e0172091. 10.1371/journal.pone.0172091. 28234931 10.1371/journal.pone.0172091 PMC5325474

[24] Packer RMA, O’Neill DG, Fletcher F, Farnworth MJ. Come for the looks, stay for the personality? A mixed methods investigation of reacquisition and owner recommendation of Bulldogs, French Bulldogs and Pugs. PLoS One. 2020;15(8):e0237276. 10.1371/journal.pone.0237276. 32845902 10.1371/journal.pone.0237276 PMC7449392

[25] Französische Bulldoggen Verein Deutschland e.V. Aktueller Rassestandard für die Französische Bulldogge. Available from: https://fbvd.de/unsere-rasse/rassestandard/. Accessed on date 2025 Jan 21.

[26] Bundesrecht konsolidiert. RIS - Tierschutzgesetz. 2025. Available from: https://www.ris.bka.gv.at/GeltendeFassung.wxe?Abfrage=Bundesnormen&Gesetzesnummer=20003541. Accessed on date 2025 Jan 21.

[27] Bundesamt für Lebensmittelsicherheit und Veterinärwesen (BLV). Tierschutz. Available from: https://www.blv.admin.ch/blv/de/home/tiere/tierschutz.html. Accessed on date 2025 Jan 21.

[28] Limb M. Dutch crackdown on brachycephalic breeds. Vet Rec. 2019;184(23):693. Available from: 10.1136/vr.l4071.

[29] BMEL. Bundestag berät über Tierschutzgesetz. 2024. Available from: https://www.bmel.de/SharedDocs/Meldungen/DE/Presse/2024/240926-tierschutzgesetz-bt.html. Accessed on date 2025 Jan 15.

[30] Ladlow J, Liu NC, Kalmar L, Sargan D. Brachycephalic obstructive airway syndrome. Vet Rec. 2018;182(13):375. 10.1136/vr.k1403. 29599258 10.1136/vr.k1403

[31] The Kennel Club. French Bulldog. *Breeds A to Z.* Available from: https://www.thekennelclub.org.uk/search/breeds-a-to-z/breeds/utility/french-bulldog/. Accessed on date 2024 Dec 18.

[32] Reich L, Hartnack S, Fitzi-Rathgen J, Reichler IM. Lebenserwartung meso-, dolicho- und brachyzephaler Hunderassen in der Schweiz. Schweiz Arch Tierheilkd. 2023;165:235–249. Available from: 10.17236/sat00390. 10.17236/sat00390 37021744

[33] O’Neill DG, Baral L, Church DB, Brodbelt DC, Packer RMA. Demography and disorders of the French Bulldog population under primary veterinary care in the UK in 2013. Canine Genet Epidemiol. 2018;5(1):3. 10.1186/s40575-018-0057-9. 29750111 10.1186/s40575-018-0057-9 PMC5932866

[34] Inoue M, Kwan NCL, Sugiura K. Estimating the life expectancy of companion dogs in Japan using pet cemetery data. J Vet Med Sci. 2018;80(7):1153–8. 10.1292/jvms.17-0384. 29798968 10.1292/jvms.17-0384 PMC6068313

[35] Statista. Japan - religious affiliation. 2021. Available from: https://www.statista.com/statistics/237609/religions-in-japan/. Accessed on date 2025 Feb 5.

[36] Kogure N, Yamazaki K. Attitudes to animal euthanasia in Japan: a brief review of cultural influences. Anthrozoos. 1990;3(3):151–4. 10.2752/089279390787057559.

[37] Sugita H, Irimajiri M. A survey of veterinarians’ attitudes toward euthanasia of companion animals in Japan. Anthrozoos. 2016;29(2):297–310. 10.1080/08927936.2016.1152722.

[38] Rosser EJ. Diagnosis of food allergy in dogs. J Am Vet Med Assoc. 1993;203(2):259–62. 10.2460/javma.1993.203.02.259. 8407485

[39] Picco F, Zini E, Nett C, Naegeli C, Bigler B, Rüfenacht S, et al. A prospective study on canine atopic dermatitis and food-induced allergic dermatitis in Switzerland. Vet Dermatol. 2008;19(3):150–5. 10.1111/j.1365-3164.2008.00669.x. 18477331 10.1111/j.1365-3164.2008.00669.x

[40] Dong Y, Wang L, Zhang K, Zhang H, Guo D. Prevalence and association with environmental factors and establishment of prediction model of atopic dermatitis in pet dogs in China. Front Vet Sci. 2024;11:1428805. 10.3389/fvets.2024.1428805. 39386248 10.3389/fvets.2024.1428805 PMC11461458

[41] Farrell LL, Schoenebeck JJ, Wiener P, Clements DN, Summers KM. The challenges of pedigree dog health: approaches to combating inherited disease. Canine Genet Epidemiol. 2015;2(1):3. 10.1186/s40575-015-0014-9. 26401331 10.1186/s40575-015-0014-9 PMC4579364

[42] Corbee RJ, Woldring HH, van den Eijnde LM, Wouters EGH. A Cross-Sectional Study on Canine and Feline Anal Sac Disease. Animals. 2022;12(1):95. Available from: 10.3390/ani12010095. 10.3390/ani12010095 PMC8749694 35011201

[43] Evans KM, Adams VJ. Proportion of litters of purebred dogs born by caesarean section. J Small Anim Pract. 2010;51(2):113–8. 10.1111/j.1748-5827.2009.00902.x. 20136998 10.1111/j.1748-5827.2009.00902.x

[44] Gobello C, de la Sota RL, Goya RG. A review of canine pseudocyesis. Reprod Domest Anim. 2001;36(6):283–8. 10.1046/j.1439-0531.2001.00322.x. 11928922 10.1046/j.1439-0531.2001.00322.x

[45] Gobello C. Revisiting canine pseudocyesis. Theriogenology. 2021;167:94–8. 10.1016/j.theriogenology.2021.03.014. 33799011 10.1016/j.theriogenology.2021.03.014

[46] Root AL, Parkin TD, Hutchison P, Warnes C, Yam PS. Canine pseudopregnancy: an evaluation of prevalence and current treatment protocols in the UK. BMC Vet Res. 2018;14(1):170. 10.1186/s12917-018-1493-1. 29793494 10.1186/s12917-018-1493-1 PMC5968611

[47] Falceto MV, Garrido AM, Bueno M, Tejedor MT, Cantin J, Garza-Moreno L, et al. Prevalence of pseudopregnancy in bitch attending a veterinary teaching hospital in Spain. Reprod Domest Anim. 2024;59(Suppl 3):e14638. 10.1111/rda.14638. 39396859 10.1111/rda.14638

[48] Trevejo R, Yang M, Lund EM. Epidemiology of surgical castration of dogs and cats in the United States. J Am Vet Med Assoc. 2011;238(7):898–904. 10.2460/javma.238.7.898. 21453178 10.2460/javma.238.7.898

[49] Brodey RS, Fidler IJ, Howson AE. The relationship of estrous irregularity, pseudopregnancy, and pregnancy to the development of canine mammary neoplasms. J Am Vet Med Assoc. 1966;149(8):1047–9. 6008416

[50] Morris JS, Dobson JM, Bostock DE, O’Farrell E. Effect of ovariohysterectomy in bitches with mammary neoplasms. Vet Rec. 1998;142(24):656–8. Available from: 10.1136/vr.142.24.656. 10.1136/vr.142.24.656 9670443

[51] Varney D, O’Neill D, O’Neill M, Church D, Stell A, Beck S et al. Epidemiology of mammary tumours in bitches under veterinary care in the UK in 2016. Vet Rec. 2023;193(5):e3054. Available from: 10.1002/vetr.3054. 10.1002/vetr.3054 37231594

[52] Oliveira L, Oliveira R, Loretti A, Rodrigues R, Driemeier D. Aspectos epidemiológicos Da neoplasia mamária Canina. Acta Sci Vet. 2018;31:105.

[53] Silva HC, Oliveira AR, Horta RS, Merísio ACR, Sena BV, Souza MCC et al. Epidemiology of canine mammary gland tumours in Espírito Santo, Brazil. Acta Sci Vet. 2019;47. Available from: 10.22456/1679-9216.89901.

[54] Fidler IJ, Brodey RS, Howson AE, Cohen D. Relationship of estrous irregularity, pseudopregnancy, and pregnancy to canine pyometra. J Am Vet Med Assoc. 1966;149(8):1043–6. 6008415

[55] Whitney JC. The pathology of the canine genital tract in false pregnancy. J Small Anim Pract. 1967;8(5):247–63. Available from:. 6068975 10.1111/j.1748-5827.1967.tb04549.x

[56] Hagman R, Lagerstedt AS, Hedhammar Å, Egenvall A. A breed-matched case-control study of potential risk factors for canine pyometra. Theriogenology. 2011;75(7):1251–7. 10.1016/j.theriogenology.2010.11.038. 21196041 10.1016/j.theriogenology.2010.11.038

[57] Niskanen JE, Reunanen V, Salonen M, Bannasch D, Lappalainen AK, Lohi H, et al. Canine DVL2 variant contributes to brachycephalic phenotype and caudal vertebral anomalies. Hum Genet. 2021;140(11):1535–45. 10.1007/s00439-021-02261-8. 33599851 10.1007/s00439-021-02261-8 PMC8519842

[58] Dupre G, Findji L, Oechtering G. Brachycephalic airway syndrome. In: Monnet E, editor. Small Anim soft tissue Surg. Hoboken, NJ: Wiley-Blackwell; 2013. pp. 167–83.

[59] Hall EJ, Carter AJ, Bradbury J, Barfield D, O’Neill DG. Proposing the vetcompass clinical grading tool for heat-related illness in dogs. Sci Rep. 2021;11(1):6828. 10.1038/s41598-021-86235-w. 33767275 10.1038/s41598-021-86235-w PMC7994647

[60] Meola SD. Brachycephalic airway syndrome. Top Companion Anim Med. 2013;28(3):91–6. 10.1053/j.tcam.2013.06.004. 24182996 10.1053/j.tcam.2013.06.004

[61] van der Gaag I, Toorenburg JV, Voorhout G, Happe RP, Aalfs RHG. Histiocytic ulcerative colitis in a French bulldog. J Small Anim Pract. 1978;19(1–12):283–90. 10.1111/j.1748-5827.1978.tb05493.x. 661239 10.1111/j.1748-5827.1978.tb05493.x

[62] Tanaka H, Nakayama M, Takase K. Histiocytic ulcerative colitis in a French bulldog. J Vet Med Sci. 2003;65(3):431–3. 10.1292/jvms.65.431. 12679583 10.1292/jvms.65.431

[63] Conrado FO, Jones EA, Graham EA, Simpson KW, Craft WF, Beatty SSK. Cytologic, histopathologic, and clinical features of granulomatous colitis in a French bulldog. Vet Clin Pathol. 2022;50(Suppl 1):76–82. 10.1111/vcp.12944. 33942344 10.1111/vcp.12944

[64] Nolte A, Junginger J, Baum B, Hewicker-Trautwein M. Heterogeneity of macrophages in canine histiocytic ulcerative colitis. Innate Immun. 2017;23(3):228–39. 10.1177/1753425916686170. 28100085 10.1177/1753425916686170

[65] Perego R, Proverbio D, Roccabianca P, Spada E. Color Dilution alopecia in a blue doberman pinscher crossbreed. Can Vet J. 2009;50(5):511–4. PMID: 19436637IF: 1.0 Q3 B4. 19436637 PMC2671874

[66] Beco L, Fontaine J, Gross TL, Charlier G. Colour dilution alopecia in seven Dachshunds. A clinical study and the hereditary, microscopical and ultrastructural aspect of the disease. Vet Dermatol. 1996;7(2):91–7. 10.1111/j.1365-3164.1996.tb00232.x. 34645045 10.1111/j.1365-3164.1996.tb00232.x

[67] Kim JH, Kang KI, Sohn HJ, Woo GH, Jean YH, Hwang EK. Color-dilution alopecia in dogs. J Vet Sci. 2005;6(3):259–61. PMID: 16131833IF: 1.5 Q2 B3. 16131833

[68] TierSchG. Tierschutzgesetz. Available from: https://www.gesetze-im-internet.de/tierschg/BJNR012770972.html. Accessed on date 2025 Jan 14.

[69] Bannasch D, Famula T, Donner J, Anderson H, Honkanen L, Batcher K, et al. The effect of inbreeding, body size and morphology on health in dog breeds. Canine Med Genet. 2021;8(1):12. 10.1186/s40575-021-00111-4. 34852838 10.1186/s40575-021-00111-4 PMC8638537

[70] Ghirlanda S, Acerbi A, Herzog H, Serpell JA. Fashion vs. Function in Cultural Evolution: The Case of Dog Breed Popularity. Bentley RA, editor. PLoS One. 2013;8(9):e74770. Available from: 10.1371/journal.pone.0074770. 10.1371/journal.pone.0074770 PMC3770587 24040341

