# Supplementary material for: Health status and disease prevalences in French bulldogs in Germany: insights from a survey-based study
Source: Companion Anim Health Genet. 2025 Oct 31;12:9. doi: 10.1186/s40575-025-00149-8 (PMC12577395; doi:10.1186/s40575-025-00149-8)
Supplement: Supplementary file 1 — Supplementary Material 1. [file 40575_2025_149_MOESM1_ESM.pdf]

The survey takes about 15 minutes to complete.

You will be asked general and specific questions about the organ systems of your French Bulldog. If your dog is not or has not been affected by a disease, please select "*no answer*". If you do not know the exact age of your dog at the time of illness, you can estimate. It is possible and even desirable to take part in the survey even if your dog has already died.

Only complete surveys can be considered.

If you own several French Bulldogs, please complete this survey for each dog separately.

Section A: General questions

In this complex you will be asked general questions about your French Bulldog.

A1.

What is your dog's name?

Answering is optional

A2.

Has your dog already passed away?

If yes, please fill out the rest of the survey regarding that dog.

Yes ☐

No ☐

A3. **How old was your dog, when he or she passed away?**

What was the cause of death of your dog?

e.g. liver tumor, epileptic seizure, sepsis due to (...)

A4.

When was your French Bulldog born?

If you don't know the exact date, you can guess.

A5. **What gender is your dog?**

Female ☐

Male ☐

A6.

Is your dog neutered?

uncastrated ☐

surgically neutered/spayed ☐

hormone-treated/chipped ☐

A7. **If your dog has been treated for health reasons , please state the reason.**

LimeSurvey

A8.

Is your French Bulldog purebred?

For the sake of simplicity, a French Bulldog is referred to as purebred if its parents are proven to be French Bulldogs OR possess the essential characteristics of the breed.

- Yes, verifiable (e.g. breeding papers of the parents) ☐
- Yes, most likely by appearance (but no papers) ☐
- I don't know ☐
- No ☐

A9.

What other breed or breeds are in your French Bulldog?

e.g.: Pug, Boston Terrier

Father: ☐☐☐☐☐☐☐☐☐☐

Mother: ☐☐☐☐☐☐☐☐☐☐

A10. Is your dog used for breeding?

- Yes ☐
- No ☐

A11.

How tall is your dog <u>in cm?</u>

The size is measured from the withers to the ground

☐☐☐☐☐☐☐☐☐☐

A12. How much does your dog weigh in <u>kg?</u>

☐☐☐☐☐☐☐☐☐☐

A13.

What color coat does your dog have?

fawn red fawn spotted/pied black brindle

- [ ] fawn
- [ ] red fawn
- [ ] spotted/pied
- [ ] black
- [ ] cream
- [ ] brindle
- [ ] white
- [ ] blue
- [ ] lilac
- [ ] tan
- [ ] Merle
- [ ] Other

Other

A14.

Mark the statements that apply to your French Bulldog!

- [ ] my dog is fluffy (long-haired)
- [ ] my dog carries the Merle gene (special coloring)
- [ ] my dog is breathing freely
- [ ] my dog is a retro Frenchie/Bulldog (back breeding with a longer nose, usually also a long tail)
- [ ] my dog has white ears
- [ ] None of the above

A15. **How did you get your dog?**

- from a breeding ☐
- from the animal shelter ☐
- from acquaintances/friends ☐
- from animal protection ☐
- Online Forums/eBay ☐
- Other ☐

Other

A16. **My dog is regularly...**

- vaccinated ☐
- dewormed ☐
- treated against fleas and ticks ☐
- none of the above ☐
- Other ☐

Other

A17.

If your dog needs to take medication regularly, please state which ones.

LimeSurvey

A18. **Does your French Bulldog have health insurance?**

- no ☐
- only surgery insured ☐
- fully insured ☐

A19. **Which country does your dog come from?**

- Germany ☐
- Switzerland ☐
- Austria ☐
- Spain ☐
- Bulgaria ☐
- Hungary ☐
- Romania ☐
- USA ☐
- England ☐
- Australia ☐
- Other ☐

Other

Section B: Digestive organs

You will be asked questions about your dog's digestive organs. These include the oral cavity, esophagus, stomach and intestinal tract, as well as the liver and pancreas.

Please click on "no answer" if your dog does not have the respective disease.

B1.

At what age was your French Bulldog diagnosed with the following diseases?

If your dog is not sick, select "no answer"

If your dog was cured and has fallen ill again, please also mark the age at the time of the return of the illness!

	<1 year	1-2 years	3-4 years	5-6 years	7-8 years	9-10 years	11-12 years	13-14 years	>14 years
tartar	☐	☐	☐	☐	☐	☐	☐	☐	☐
stomatitis (inflammation of the gums)	☐	☐	☐	☐	☐	☐	☐	☐	☐
underbite (teeth of the lower jaw in front of the teeth of the upper jaw)	☐	☐	☐	☐	☐	☐	☐	☐	☐
missing teeth	☐	☐	☐	☐	☐	☐	☐	☐	☐
persistent milk teeth (milk teeth have not fallen out)	☐	☐	☐	☐	☐	☐	☐	☐	☐
megaesophagus (enlargement of the esophagus diameter)	☐	☐	☐	☐	☐	☐	☐	☐	☐
pyloric stenosis (gastric emptying disorder)	☐	☐	☐	☐	☐	☐	☐	☐	☐
histiocytic ulcerative colitis (boxer colitis/colon disease)	☐	☐	☐	☐	☐	☐	☐	☐	☐
diaphragmatic hernia (rupture of the diaphragm)	☐	☐	☐	☐	☐	☐	☐	☐	☐
food allergy/incompatibility	☐	☐	☐	☐	☐	☐	☐	☐	☐
pancreatitis (inflammation of the pancreas)	☐	☐	☐	☐	☐	☐	☐	☐	☐
portosystemic shunt (faulty vascular connection)	☐	☐	☐	☐	☐	☐	☐	☐	☐
IBD/CIE (inflammatory bowel disease)	☐	☐	☐	☐	☐	☐	☐	☐	☐
PLE (protein loss syndrome)	☐	☐	☐	☐	☐	☐	☐	☐	☐
hemorrhagic gastroenteritis (acute bloody diarrhea)	☐	☐	☐	☐	☐	☐	☐	☐	☐

LimeSurvey

	<1 year	1-2 years	3-4 years	5-6 years	7-8 years	9-10 years	11-12 years	13-14 years	>14 years
anal sac inflammation/drainage disorder	☐	☐	☐	☐	☐	☐	☐	☐	☐
hepatitis (liver inflammation)	☐	☐	☐	☐	☐	☐	☐	☐	☐

B2.

Which foods is your dog allergic to?

- poultry/chicken ☐
- Grain ☐
- Beef ☐
- Other ☐

Other

B3. **How does your dog's food allergy manifest itself?**

- increased itching ☐
- licking the paws ☐
- ear problems ☐
- diarrhea ☐
- heartburn/belching/reflux ☐
- flatulence/bloating ☐

B4.

Has your dog ever been diagnosed with any of the following infectious diseases?

If your dog was cured and has fallen ill again, please also mark the age at the time of the return of the illness!

	<1 year	1-2 years	3-4 years	5-6 years	7-8 years	9-10 years	11-12 years	13-14 years	>14 years
parvovirus (infection with the parvovirus, mostly in puppies, bloody diarrhea)	☐	☐	☐	☐	☐	☐	☐	☐	☐
giardia (parasites of the stomach, diarrhea)	☐	☐	☐	☐	☐	☐	☐	☐	☐

	<1 year	1-2 years	3-4 years	5-6 years	7-8 years	9-10 years	11-12 years	13-14 years	>14 years
bacterial enteritis	☐	☐	☐	☐	☐	☐	☐	☐	☐

B5.

Has your dog been diagnosed with any other diseases affecting the digestive tract (oral cavity, esophagus, stomach, intestines, anal sacs, liver, pancreas) not mentioned here?

e.g. cleft palate, gastric torsion, intestinal obstruction

Section C: respiratory system

In the following section, you will be asked questions about your dog's respiratory system. This includes the following organs: the nose, the throat, the larynx, the trachea, the pulmonary branches and the lungs.

C1. Has your dog ever shown the following symptoms?

- snoring ☐
- heat stress (excessive panting to shortness of breath during hot periods) ☐
- performance intolerance ☐
- panting even at rest ☐
- permanently audible breathing ☐
- shortness of breath ☐
- Upright position when sleeping/sitting ☐

C2.

Match your dog's nose to the following pictures!

- The nostrils are almost closed, there is hardly any space visible between the lateral and middle nostril wall ☐
- The nostrils are mostly closed, a small space between the lateral and middle nostril wall is visible ☐
- The lateral nostril wall touches the middle nostril wall at the upper part of the nostril, and the nostrils are only open downwards ☐
- Slightly narrowed nostrils where the lateral nostril wall does not touch the middle nostril wall ☐
- Wide open nostrils ☐

LimeSurvey

C3.

Match the nose length of your dog to the following pictures!

- 1 ☐
- 2 ☐
- 3 ☐
- 4 ☐
- 5 ☐
- longer ☐

C4.

At what age was your dog diagnosed with the following diseases?

If your dog was cured and has fallen ill again, please also mark the age at the time of the return of the illness!

	<1 year	1-2 years	3-4 years	5-6 years	7-8 years	9-10 years	11-12 years	13-14 years	>14 years
laryngeal collapse	☐	☐	☐	☐	☐	☐	☐	☐	☐
elongated soft palate	☐	☐	☐	☐	☐	☐	☐	☐	☐
tracheal collapse	☐	☐	☐	☐	☐	☐	☐	☐	☐
hypoplastic trachea (congenital narrowing of the trachea)	☐	☐	☐	☐	☐	☐	☐	☐	☐
BOAS (brachycephalic obstructive airway syndrome)	☐	☐	☐	☐	☐	☐	☐	☐	☐
chronic bronchitis	☐	☐	☐	☐	☐	☐	☐	☐	☐
aspiration pneumonia (pneumonia caused by inhaled components)	☐	☐	☐	☐	☐	☐	☐	☐	☐

C5.

At what age was your dog diagnosed with the following infectious diseases?

If your dog was cured and has fallen ill again, please also mark the age at the time of the return of the illness!

	<1 year	1-2 years	3-4 years	5-6 years	7-8 years	9-10 years	11-12 years	13-14 years	>14 years
lungworm infection	☐	☐	☐	☐	☐	☐	☐	☐	☐

LimeSurvey

	<1 year	1-2 years	3-4 years	5-6 years	7-8 years	9-10 years	11-12 years	13-14 years	>14 years
bacterial pneumonia (pneumonia caused by bacteria)	☐	☐	☐	☐	☐	☐	☐	☐	☐
aspergillosis (pneumonia caused by fungi)	☐	☐	☐	☐	☐	☐	☐	☐	☐
viral pneumonia (kennel cough)	☐	☐	☐	☐	☐	☐	☐	☐	☐

C6.

Has your French Bulldog ever had at least one brachycephalic surgery?

e.g. widening of the nostrils, shortening of the soft palate

- Yes, once ☐
- Yes twice ☐
- Yes, three times ☐
- not yet, but in planning ☐
- no ☐

C7.

What procedures were performed during the <u>first operation</u>?

- enlargement of the nostrils ☐
- shortening the soft palate ☐
- thinning of the soft palate ☐
- removal of the laryngeal pouches ☐
- removal of parts of the nasal turbinates ☐
- removal of the tonsils ☐
- laryngeal dilation ☐
- I don't know ☐

C8.

What procedures were performed during the <u>second operation</u>?

- enlargement of the nostrils ☐
- shortening the soft palate ☐
- thinning of the soft palate ☐
- removal of the laryngeal pouches ☐

removal of parts of the nasal turbinates ☐

removal of the tonsils ☐

laryngeal dilation ☐

I don't know ☐

C9.

Has your dog been diagnosed with any other diseases not mentioned here that affect the respiratory tract (nose, throat, larynx, trachea, pulmonary branches, lungs)?

e.g. blockage of the airways

Section D: genital organs

You will be asked questions about your dog's sexual organs.

D1. Has your dog ever had puppies?

Yes ☐

No ☐

D2. How many puppies did your dog have in each litter?

	1-2	3-4	5-6	7-8	9-10	>10
1st litter	☐	☐	☐	☐	☐	☐
2nd litter	☐	☐	☐	☐	☐	☐
3rd litter	☐	☐	☐	☐	☐	☐
4th litter	☐	☐	☐	☐	☐	☐
5th litter	☐	☐	☐	☐	☐	☐

D3. **Has your dog ever had <u>complications</u> while giving birth?**

- Yes ☐
- No ☐

D4.

What <u>complications</u> occurred during or after birth?

- prolonged birth ☐
- weak contractions ☐
- puppies getting stuck in the birth canal ☐
- stillbirths ☐
- problems in freeing the puppies from the amniotic sacs ☐
- lack of care for the puppies after birth ☐
- death of puppies during birth ☐
- mummification of the puppies ☐
- missing milk ☐
- endometritis (inflammation of the uterus) ☐
- mastitis (inflammation of the mammary glands) ☐
- puerperal tetany (calcium deficiency after birth) ☐
- Other ☐

Other

D5. **Has your dog ever had to undergo a cesarean section?**

- Yes ☐
- No ☐

D6.

Has your dog been diagnosed with a disorder of sexual development?

e.g.: Late sexual maturity

- Yes ☐
- No ☐

D7.

Has your dog ever been diagnosed with undescended testicles?

One or two testicles not descended into the scrotum

- Yes ☐
- No ☐

D8.

Which testicle was affected?

- left testicle ☐
- right testicle ☐
- both testicles ☐

D9.

At what age were the following diseases diagnosed in your French Bulldog?

If your dog was cured and has fallen ill again, please also mark the age at the time of the return of the illness!

	<1 year	1-2 years	3-4 years	5-6 years	7-8 years	9-10 years	11-12 years	13-14 years	>14 years
pseudopregnancy	☐	☐	☐	☐	☐	☐	☐	☐	☐
pyometra (uterine infection)	☐	☐	☐	☐	☐	☐	☐	☐	☐

LimeSurvey

D10.

Has your dog been diagnosed with any other diseases/problems related to the reproductive organs or fertility or birth of your dog?

Section E: urinary organs

In this group of questions you will be asked questions about your dog's urinary organs. These include: the kidneys, the ureters, the urinary bladder and the urethra.

E1.

At what age was your French Bulldog diagnosed with the following diseases?

If your dog was cured and has fallen ill again, please also mark the age at the time of the return of the illness!

	<1 year	1-2 years	3-4 years	5-6 years	7-8 years	9-10 years	11-12 years	13-14 years	>14 years
renal dysplasia (improper development of kidney tissue)	☐	☐	☐	☐	☐	☐	☐	☐	☐
chronic renal failure (limited renal function)	☐	☐	☐	☐	☐	☐	☐	☐	☐
acute renal failure (e.g. due to toxins)	☐	☐	☐	☐	☐	☐	☐	☐	☐
leptospirosis (infection with leptospira)	☐	☐	☐	☐	☐	☐	☐	☐	☐
ectopic ureter (malformed ureter)	☐	☐	☐	☐	☐	☐	☐	☐	☐
cystitis	☐	☐	☐	☐	☐	☐	☐	☐	☐
urinary stones	☐	☐	☐	☐	☐	☐	☐	☐	☐

E2. **Which urinary stones were diagnosed?**

- struvite ☐
- calcium oxalate ☐
- silicates ☐
- cystine ☐
- urates ☐

LimeSurvey

E3.

Has your dog been diagnosed <u>with other diseases</u> affecting the urinary tract?

e.g. pyelonephritis

Section F: skeletal system

You will be asked questions about your dog's skeletal system, including bones, muscles, joints, ligaments and tendons.

F1.

Has your French Bulldog ever been diagnosed <u>with wedge vertebrae</u> ?

Deformation of the vertebral bodies, which can twist the spine

	<1 year	1-2 years	3-4 years	5-6 years	7-8 years	9-10 years	11-12 years	13-14 years	>14 years
age at diagnosis	☐	☐	☐	☐	☐	☐	☐	☐	☐

F2. **Which subtype was diagnosed? *(Source: ourhumananatomy.blogspot.com)***

semi-

segmented segmented wedge-shaped kissing spine kissing-spine with wedge

- semi-segmented ☐
- segmented ☐
- wedge-shaped ☐
- kissing spine ☐
- kissing spine with hemivertebrae ☐
- I don't know ☐

Other

Other

F3.

At what age was your French Bulldog diagnosed with the following diseases?

	<1 year	1-2 years	3-4 years	5-6 years	7-8 years	9-10 years	11-12 years	13-14 years	>14 years
malformations of the skull (e.g. open fontanelle/skullcap)	☐	☐	☐	☐	☐	☐	☐	☐	☐
humeral intracondylar fissures (cracks in the humerus)	☐	☐	☐	☐	☐	☐	☐	☐	☐
hypertrophic osteodystrophy (swelling of the anterior ankle joints)	☐	☐	☐	☐	☐	☐	☐	☐	☐
HD (hip dysplasia/maldevelopment of the hip joint)	☐	☐	☐	☐	☐	☐	☐	☐	☐
ED (elbow dysplasia/ maldeveloped elbow joint)	☐	☐	☐	☐	☐	☐	☐	☐	☐
patellar luxation (paella popping out/jumping)	☐	☐	☐	☐	☐	☐	☐	☐	☐
arthrosis	☐	☐	☐	☐	☐	☐	☐	☐	☐
cruciate ligament tear	☐	☐	☐	☐	☐	☐	☐	☐	☐
spondylosis	☐	☐	☐	☐	☐	☐	☐	☐	☐
immune-related polyarthritis (rheumatism)	☐	☐	☐	☐	☐	☐	☐	☐	☐

F4. **Does your French Bulldog have a <u>shortened or missing tail</u> ?**

- ☐ Anuria (missing tail)
- ☐ Brachyuria (short tail)
- ☐ long tail
- ☐ deformed longer tail/corkscrew tail
- ☐ Other

Other

[ ]

F5.

Has your dog been diagnosed with other diseases not mentioned here that affect bones, muscles, tendons, ligaments or joints?

e.g. muscle diseases

[ ]

Section G: nervous system

You will be asked questions about your brain, spinal cord and nerves.

G1.

Has your French Bulldog ever been diagnosed with a <u>herniated disc/ IVDD</u> ?

Symptoms: Paralysis of the limbs, painful spine

	<1 year	1-2 years	3-4 years	5-6 years	7-8 years	9-10 years	11-12 years	13-14 years	>14 years
age at diagnosis	☐	☐	☐	☐	☐	☐	☐	☐	☐
age at healing	☐	☐	☐	☐	☐	☐	☐	☐	☐
age at healing (surgery)	☐	☐	☐	☐	☐	☐	☐	☐	☐
age at recurrence	☐	☐	☐	☐	☐	☐	☐	☐	☐

LimeSurvey

G2.

In which section of the spine was the disc herniation located?

- [ ] cervical (neck area)
- [ ] thoracic (thoracic spine area)
- [ ] lumbar (lumbar region)

G3. **At what age was your dog diagnosed with the following diseases?**

	<1 year	1-2 years	3-4 years	5-6 years	7-8 years	9-10 years	11-12 years	13-14 years	>14 years
meningoencephalitis (inflammation of the brain and meninges)	☐	☐	☐	☐	☐	☐	☐	☐	☐
fibrinoid leukodystrophy (degeneration of the brain and spinal cord)	☐	☐	☐	☐	☐	☐	☐	☐	☐
spinal subarachnoid diverticula (dilations that compress the spinal cord)	☐	☐	☐	☐	☐	☐	☐	☐	☐
epilepsy	☐	☐	☐	☐	☐	☐	☐	☐	☐
dementia	☐	☐	☐	☐	☐	☐	☐	☐	☐

G4.

Has your dog been diagnosed with other nervous system diseases not mentioned here?

e.g. hydrocephalus, paralysis, vestibular syndrome

Section H: eye

In this series of questions, you will be asked questions about your French bulldog's eyes. At the end, you can add any diseases not listed yourself.

H1.

Has your French Bulldog ever been diagnosed with the following eye conditions?

If your dog was cured and has fallen ill again, please also mark the age at the time of the return of the illness!

	<1 year	1-2 years	3-4 years	5-6 years	7-8 years	9-10 years	11-12 years	13-14 years	15-16 years
exophthalamus (protrusion of the eye from the eye socket)	☐	☐	☐	☐	☐	☐	☐	☐	☐
entropion (eyelid rolled towards the eye)	☐	☐	☐	☐	☐	☐	☐	☐	☐
cataract (clouding of the lens)	☐	☐	☐	☐	☐	☐	☐	☐	☐
corneal ulcer (injury to the anterior skin of the eye)	☐	☐	☐	☐	☐	☐	☐	☐	☐
canine multifocal retinopathy (retinal damage)	☐	☐	☐	☐	☐	☐	☐	☐	☐
glaucoma	☐	☐	☐	☐	☐	☐	☐	☐	☐
blindness	☐	☐	☐	☐	☐	☐	☐	☐	☐
conjunctivitis	☐	☐	☐	☐	☐	☐	☐	☐	☐
foreign body in the eye	☐	☐	☐	☐	☐	☐	☐	☐	☐

H2.

Has your dog been diagnosed with any other eye diseases not mentioned here?

Section I: ear

I1.

At what age was your French Bulldog diagnosed with the following diseases?

If your dog was cured and has fallen ill again, please also mark the age at the time of the return of the illness!

	<1 year	1-2 years	3-4 years	5-6 years	7-8 years	9-10 years	11-12 years	13-14 years	>14 years
otitis externa (inflammation of the external auditory canals)	☐	☐	☐	☐	☐	☐	☐	☐	☐
otitis media (inflammation of the middle ear canals)	☐	☐	☐	☐	☐	☐	☐	☐	☐
narrowed ear canal	☐	☐	☐	☐	☐	☐	☐	☐	☐
deafness	☐	☐	☐	☐	☐	☐	☐	☐	☐

I2.

Does your dog suffer from other ear diseases?

Section J: blood system

The following group of questions deals with diseases of the blood system. These include blood cells (red and white blood cells), blood coagulation, the vascular system, the spleen, the bone marrow, the heart and immune deficiencies.

J1. **At what age was your French Bulldog diagnosed with the following diseases?**

	<1 year	1-2 years	3-4 years	5-6 years	7-8 years	9-10 years	11-12 years	13-14 years	>14 years
pulmonary stenosis (narrowing of the drainage pathway from the right ventricle to the lungs)	☐	☐	☐	☐	☐	☐	☐	☐	☐
cardiac arrhythmia	☐	☐	☐	☐	☐	☐	☐	☐	☐

LimeSurvey

	<1 year	1-2 years	3-4 years	5-6 years	7-8 years	9-10 years	11-12 years	13-14 years	>14 years
ventricular septal defect (hole in the heart wall)	☐	☐	☐	☐	☐	☐	☐	☐	☐
mitral valve endocardiosis (thickening of the heart valves)	☐	☐	☐	☐	☐	☐	☐	☐	☐
Von Willebrand disease (bleeding disorder)	☐	☐	☐	☐	☐	☐	☐	☐	☐
hemophilia A or B (blood clotting disorder)	☐	☐	☐	☐	☐	☐	☐	☐	☐
high blood pressure	☐	☐	☐	☐	☐	☐	☐	☐	☐
autoimmune disease	☐	☐	☐	☐	☐	☐	☐	☐	☐
environmental allergy	☐	☐	☐	☐	☐	☐	☐	☐	☐

J2.

When was your dog diagnosed with the following infectious diseases ?

If your dog was cured and has become ill again , please also tick the age at the time of re-illness !

leishmaniasis ☐

babesiosis (canine malaria) ☐

heartworm ☐

J3. **Has your dog been diagnosed with other diseases affecting the bone marrow, spleen, heart, blood cells, immune deficiencies or blood vessels?**

Section K: skin

In this questionnaire you will be asked 3 questions about your dog's skin and hair.

K1.

At what age was your dog diagnosed with the following diseases?

If your dog was <u>cured</u> and has fallen i<u>ll again</u>, please also mark the age at the time of the <u>return</u> of the illness!

	<1 year	1-2 years	3-4 years	5-6 years	7-8 years	9-10 years	11-12 years	13-14 years	>14 years
skin fold dermatitis (inflammation of the skin folds)	☐	☐	☐	☐	☐	☐	☐	☐	☐
atopic dermatitis (eczema)	☐	☐	☐	☐	☐	☐	☐	☐	☐
chin pyoderma (acne)	☐	☐	☐	☐	☐	☐	☐	☐	☐
demodicosis (caused by Demodex mites)	☐	☐	☐	☐	☐	☐	☐	☐	☐
color mutant alopecia (thinning of the fur due to certain coat colors)	☐	☐	☐	☐	☐	☐	☐	☐	☐

K2. Has your dog ever shown any of the following symptoms:

- hair loss ☐
- pedunculated warts ☐
- purulent rash ☐
- small red spots ☐
- dandruff ☐
- paw licking ☐
- increased itching ☐
- hives (thickened areas, similar to mosquito bites) ☐

LimeSurvey

K3.

Has your dog had any other skin problems not mentioned here?

e.g. skin cancer, particularly high/frequent flea infestation, hives, hairless areas

Section L: hormones

In this set of questions, you will be asked about illnesses your dog has that affect the hormone balance. Hormones include: thyroid hormones, sex hormones, bone metabolism hormones, adrenal hormones, and the associated centers: hypothalamus and pituitary gland.

L1. At what age was your dog diagnosed with the following diseases?

	<1 year	1-2 years	3-4 years	5-6 years	7-8 years	9-10 years	11-12 years	13-14 years	>14 years
hypothyroidism (underactive thyroid)	☐	☐	☐	☐	☐	☐	☐	☐	☐
hypoadrenocorticism/Addison's disease (underfunction of the adrenal cortex)	☐	☐	☐	☐	☐	☐	☐	☐	☐
hyperadrenocorticism/Cushing syndrome (overactivity of the adrenal cortex)	☐	☐	☐	☐	☐	☐	☐	☐	☐
diabetes mellitus	☐	☐	☐	☐	☐	☐	☐	☐	☐

L2.

Has your dog been diagnosed with other hormone-related diseases?

e.g. Diabetes insipidus

LimeSurvey

Section M: tumors

M1.

Has your dog ever been diagnosed with any of the following tumors ?

	<1 year	1-2 years	3-4 years	5-6 years	7-8 years	9-10 years	11-12 years	13-14 years	15-16 years
melanoma (skin cancer)	☐	☐	☐	☐	☐	☐	☐	☐	☐
mast cell tumor	☐	☐	☐	☐	☐	☐	☐	☐	☐
lymphoma (cancer of the lymphatic system)	☐	☐	☐	☐	☐	☐	☐	☐	☐
osteosarcoma (bone cancer)	☐	☐	☐	☐	☐	☐	☐	☐	☐
bladder tumor	☐	☐	☐	☐	☐	☐	☐	☐	☐
liver tumor	☐	☐	☐	☐	☐	☐	☐	☐	☐
kidney tumor	☐	☐	☐	☐	☐	☐	☐	☐	☐
spleen tumor	☐	☐	☐	☐	☐	☐	☐	☐	☐
mammary tumor (mammary gland tumor)	☐	☐	☐	☐	☐	☐	☐	☐	☐
brain tumor	☐	☐	☐	☐	☐	☐	☐	☐	☐
insulinoma (tumor of the pancreas)	☐	☐	☐	☐	☐	☐	☐	☐	☐
testicular tumor	☐	☐	☐	☐	☐	☐	☐	☐	☐
bone marrow tumor/tumor of the blood system	☐	☐	☐	☐	☐	☐	☐	☐	☐

M2.

How old was your dog when it healed ? Were there any recurrences ?

	<1 year	1-2 years	3-4 years	5-6 years	7-8 years	9-10 years	11-12 years	13-14 years	15-16 years	>16 years
age at healing	☐	☐	☐	☐	☐	☐	☐	☐	☐	☐

	<1 year	1-2 years	3-4 years	5-6 years	7-8 years	9-10 years	11-12 years	13-14 years	15-16 years	>16 years
age at recovery (chemotherapy)	☐	☐	☐	☐	☐	☐	☐	☐	☐	☐
age at recurrence	☐	☐	☐	☐	☐	☐	☐	☐	☐	☐

M3.

If you have further information about the tumor, you can provide it here!

e.g.: Breast tumor: benign mixed cell breast tumor / Brain tumor: oligodendroglial tumor

M4.

Has your dog ever been diagnosed with another type of tumor?

How old was your dog when diagnosed?

Section N: anesthesia

N1.

Has your dog ever been treated under general anesthesia ?

e.g. for castration, eye surgery, tooth extraction

Yes ☐

No ☐

N2. **Did one or more complications occur during or after the anesthesia?**

drop in blood pressure ☐

LimeSurvey

- [ ] increase in blood pressure
- [ ] hypothermia
- [ ] hyperthermia
- [ ] organ failure
- [ ] cardiac arrest
- [ ] allergic reaction
- [ ] drug intolerance due to ABCB-1 gene defect
- [ ] respiratory arrest
- [ ] Other

Other

Section O: behavioral disorders

O1. My dog shows....

	Joy	indifferenc e	Fear	aggression
towards strangers	☐	☐	☐	☐
towards people you know	☐	☐	☐	☐
towards strange dogs	☐	☐	☐	☐
towards familiar dogs	☐	☐	☐	☐
towards children	☐	☐	☐	☐
towards men	☐	☐	☐	☐
when alone	☐	☐	☐	☐
with loud noises (bangs)	☐	☐	☐	☐

Section P: Contact

P1.

This survey will be evaluated and published. If you are interested in the data, you can provide your e-mail address.

In some cases it may be possible that further questions arise. May I ask you questions via your e-mail address?

Your e-mail address will only be used in the context of this survey and will be deleted after completion of the doctoral thesis.

I would like to receive the results ☐

Comment

I am ready to answer any questions ☐

Comment

P2.

I hereby confirm that all information has been answered conscientiously and honestly. Any diagnoses my dog has received were made by a veterinarian.

All information provided here <u>regarding my dog</u> may be used for research purposes without restriction.

Yes ☐

No ☐

Thank you for your support!

Do you have any questions or comments?

Then please contact me at: mh53ruho@studserv.uni-leipzig.de
