# Supplementary material for: Health status and disease prevalences in French bulldogs in Germany: insights from a survey-based study
Source: Companion Anim Health Genet. 2025 Oct 31;12:9. doi: 10.1186/s40575-025-00149-8 (PMC12577395; doi:10.1186/s40575-025-00149-8)
Supplement: Supplementary file 2 — Supplementary Material 2. [file 40575_2025_149_MOESM2_ESM.docx]

Supplement 5: Most common gastrointestinal disorders in French bulldogs in this study (n=574). Comparison of disease/condition prevalence in male *vs.* female dogs.

| **Disease/condition** | **Occurrence** | **Total Preva- lence** | **Confidence Interval (CI)** | **Female Preva- lence** | **Male Preva- lence** | **p-value** |
| --- | --- | --- | --- | --- | --- | --- |
| Food allergy/ hypersensitivity  Underbite | 281  120 | 0.490  0.209 | 0.45 – 0.53  0.18 – 0.24 | 0.465  0.238 | 0.514  0.182 | .274  .121 |
| *Giardia spp.* in fecal samples | 117 | 0.204 | 0.17 – 0.24 | 0.188 | 0.219 | .409 |
| Dental calculus | 111 | 0.193 | 0.16 – 0.23 | 0.191 | 0.195 | .994 |
| Anal sac disease | 89 | 0.155 | 0.13 – 0.19 | 0.174 | 0.137 | .271 |
| Pancreatitis | 58 | 0.101 | 0.08 – 0.13 | 0.099 | 0.103 | 1.000 |
| Persistent deciduous teeth | 32 | 0.056 | 0.04 – 0.08 | 0.046 | 0.065 | .419 |
| Stomatitis | 29 | 0.051 | 0.04 – 0.07 | 0.057 | 0.045 | .633 |
| Hemorrhagic gastroenteritis | 23 | 0.040 | 0.03 – 0.06 | 0.046 | 0.034 | .609 |
| IBD/CIE* | 14 | 0.024 | 0.01 – 0.04 | 0.028 | 0.021 | .736 |
| Parvovirus infection | 12 | 0.021 | 0.01 – 0.04 | 0.021 | 0.021 | 1.000 |
| Bacterial enteritis | 9 | 0.016 | 0.01 – 0.03 | 0.021 | 0.010 | .469 |
| Pyloric stenosis | 7 | 0.012 | 0.01 – 0.02 | 0.007 | 0.017 | .475 |
| Megaesophagus | 4 | 0.007 | 0.00 – 0.02 | 0.004 | 0.010 | .641 |
| Protein-losing enteropathy (PLE) | 4 | 0.007 | 0.00 – 0.02 | 0.014 | - | .123 |
| Histiocytic ulcerative gastritis | 3 | 0.005 | 0.00 – 0.02 | 0.004 | 0.007 | 1.000 |
| Diaphragmatic hernia | 2 | 0.003 | 0.00 – 0.01 | 0.007 | - | .463 |
| Hepatitis | 2 | 0.003 | 0.00 – 0.01 | 0.007 | - | .463 |

** Inflammatory bowel disease/Chronic inflammatory enteropathy*

Supplement 6: Concurrent medical conditions of French bulldogs with adverse food reactions, including food allergy or food hypersensitivity (n=281).

| **Disease/condition** | **Food allergy** | **Prevalence** | **Confidence** | **Odds** | **p-value** |
| --- | --- | --- | --- | --- | --- |
|  | **(n=281)** |  | **Interval (CI)** | **Ratio** |  |
|  |  |  |  | **(OR)** |  |
| Atopic dermatitis | 57 | 0.203 | 0.16 - 0.25 | 5.1 | *<*.001*** |
| Constricted ear canal | 25 | 0.089 | 0.06 - 0.13 | 4.7 | *<*.001*** |
| Environmental allergy | 56 | 0.199 | 0.16 - 0.25 | 4.0 | *<*.001*** |
| Skin fold dermatitis | 83 | 0.295 | 0.25 - 0.35 | 3.7 | *<*.001*** |
| Pancreatitis | 43 | 0.153 | 0.12 - 0.20 | 3.3 | *<*.001*** |
| Otitis | 134 | 0.477 | 0.42 - 0.54 | 2.8 | *<*.001*** |
| Anal sac disease | 59 | 0.210 | 0.17 - 0.26 | 2.3 | *<*.001*** |
| Conjunctivitis | 79 | 0.281 | 0.23 - 0.34 | 2.2 | *<*.001*** |

** p < 0.05; ** p < 0.01; *** p < 0.001*

Supplement 7: **Prevalence of skin and ear conditions in the studied population (n=574) and comparison**

**of the prevalences between male *vs.* female dogs.**

| **Disease/condition** | **Occurrences** | **Total Preva- lence** | **Confidence Interval (CI)** | **Female Preva- lence** | **Male Preva- lence** | **p-value** |
| --- | --- | --- | --- | --- | --- | --- |
| Otitis externa | 165 | 0.287 | 0.25 – 0.33 | 0.312 | 0.264 | .235 |
| Skin fold dermatitis | 113 | 0.1972 | 0.17 – 0.23 | 0.177 | 0.216 | .292 |
| Otitis media | 109 | 0.190 | 0.16 – 0.22 | 0.181 | 0.199 | .662 |
| Environmental allergy | 73 | 0.127 | 0.10 – 0.16 | 0.128 | 0.127 | 1.000 |
| Atopic dermatitis | 71 | 0.124 | 0.10 – 0.15 | 0.117 | 0.130 | .726 |
| Demodicosis | 48 | 0.084 | 0.06 – 0.11 | 0.082 | 0.086 | .980 |
| Chin pyoderma | 22 | 0.038 | 0.03 – 0.06 | 0.039 | 0.038 | 1.000 |
| Color mutant alopecia | 15 | 0.026 | 0.02 – 0.04 | 0.032 | 0.021 | .554 |
|  |  |  |  |  |  |  |

Supplement 8: Respiratory disorders in French bulldogs in this study (n=574) and comparison of their prevalences in male *vs.* female dogs.

| **Disease/condition** | | **Occurrences** | **Total Preva- lence** | **Confidence Interval (CI)** | **Female Preva- lence** | **Male Preva- lence** | **p-value** |
| --- | --- | --- | --- | --- | --- | --- | --- |
| Stenotic nares | | 191 | 0.351 | 0.31 - 0.39 | 0.310 | 0.393 | .055 |
| Elongated soft palate | | 152 | 0.265 | 0.23 - 0.30 | 0.255 | 0.274 | .681 |
| BOAS* | | 77 | 0.134 | 0.11 - 0.16 | 0.110 | 0.158 | .121 |
| Hypoplastic trachea | 27 | 0.047 | 0.03 - 0.07 | 0.046 | 0.048 | 1.000 |  |
| Viral pneumonia | | 26 | 0.045 | 0.03 - 0.07 | 0.046 | 0.045 | 1.000 |
| Laryngeal collapse | | 14 | 0.024 | 0.01 - 0.04 | 0.021 | 0.027 | .838 |
| Tracheal collapse | | 7 | 0.012 | 0.01 - 0.02 | 0.014 | 0.010 | .963 |
| Aspiration pneumonia | | 6 | 0.010 | 0.00 - 0.02 | 0.007 | 0.014 | .713 |
| Lungworm infection | | 6 | 0.010 | 0.00 - 0.02 | 0.011 | 0.010 | 1.000 |
| Chronic bronchitis | | 4 | 0.007 | 0.00 - 0.02 | 0.014 | - | - |
| Bacterial pneumonia | | 1 | 0.002 | 0.00 - 0.01 | 0.004 | - | - |

**Brachycephalic obstructive airway syndrome*

Supplement 9: Prevalence of ophthalmological conditions in male *vs.* female dogs in the studied population (n=574).

| **Disease/condition** | **Occurrences** | **Total Preva- lence** | **Confidence Interval (CI)** | **Female Preva- lence** | **Male Preva- lence** | ***p*-value** |
| --- | --- | --- | --- | --- | --- | --- |
| Conjunctivitis | 124 | 0.216 | 0.18 – 0.25 | 0.199 | 0.233 | .370 |
| Corneal ulcer | 87 | 0.152 | 0.12 – 0.18 | 0.138 | 0.164 | .450 |
| Ocular foreign body | 41 | 0.071 | 0.05 – 0.10 | 0.078 | 0.065 | .660 |
| Cataract | 37 | 0.064 | 0.05 – 0.09 | 0.060 | 0.068 | .818 |
| Multifocal retinopathy | 30 | 0.052 | 0.04 – 0.07 | 0.060 | 0.045 | .509 |
| Exophthalmos | 26 | 0.045 | 0.03 – 0.07 | 0.028 | 0.062 | .086 |
| Cherry eye | 16 | 0.028 | 0.02 – 0.04 | 0.018 | 0.038 | .231 |
| Blindness | 12 | 0.021 | 0.01 – 0.04 | 0.025 | 0.017 | .724 |
| Entropion | 9 | 0.016 | 0.01 – 0.03 | 0.014 | 0.017 | 1.000 |
| Ocular dermoid | 7 | 0.012 | 0.01 – 0.02 | 0.011 | 0.014 | 1.000 |
| Glaucoma | 4 | 0.007 | 0.00 – 0.02 | 0.011 | 0.003 | .591 |

Supplement 10: Prevalence and risk of concurrent dermatological and ophthalmological conditions in dogs with conjunctivitis (n=124) in the study population

| **Disease/condition** | **Conjunctivitis**  **(n=124)** | **Preva-lence** | **Confidence Interval (CI)** | **Odds Ratio (OR)** | **p-value** |
| --- | --- | --- | --- | --- | --- |
| Skin fold dermatitis | 47 | 0.379 | 0.30 – 0.47 | 3.6 | *<*.001*** |
| Atopic dermatitis | 31 | 0.250 | 0.18 – 0.33 | 3.4 | *<*.001*** |
| Corneal ulcer | 35 | 0.282 | 0.21 – 0.37 | 3.0 | *<*.001*** |
| Exophthalmos | 11 | 0.089 | 0.05 – 0.15 | 2.8 | .012* |
| Allergy | 86 | 0.694 | 0.61 – 0.77 | 2.5 | *<*.001*** |
| BOAS | 27 | 0.218 | 0.15 – 0.30 | 2.2 | .002** |

** p < 0.05; ** p < 0.01; *** p < 0.001; BOAS: brachycephalic obstructive airway syndrome*

Supplement 11: **Prevalences and sex distribution of musculoskeletal diseases in the French Bulldogs in this study (n=574).** Tail malformations are not included in this table.

| **Disease/condition** | **Occurrences (Fraction)** | **Total Preva- lence** | **Confidence Interval (CI)** | **Female Preva- lence** | **Male Preva- lence** | ***p*-value** |
| --- | --- | --- | --- | --- | --- | --- |
| Hemivertebrae | 141 | 0.246 | 0.21–0.28 | 0.227 | 0.264 | .355 |
| Patellar luxation | 61 | 0.106 | 0.08–0.13 | 0.106 | 0.106 | 1.000 |
| Osteoarthritis | 44 | 0.077 | 0.06–0.10 | 0.078 | 0.075 | 1.000 |
| Spondylosis | 44 | 0.077 | 0.06–0.10 | 0.078 | 0.075 | 1.000 |
| Hip dysplasia (HD) | 26 | 0.045 | 0.03–0.07 | 0.060 | 0.031 | .135 |
| Cruciate ligament rupture | 10 | 0.017 | 0.01–0.03 | 0.021 | 0.014 | .708 |
| Elbow dysplasia (ED) | 8 | 0.014 | 0.01–0.03 | 0.018 | 0.010 | .685 |
| Hypertrophic osteodystrophy | 2 | 0.003 | 0.00–0.01 | 0.000 | 0.007 | .494 |
| Immune-mediated polyarthritis | 2 | 0.003 | 0.00–0.01 | 0.004 | 0.003 | 1.000 |
|  |  |  |  |  |  |  |

Supplement 12: Concurrent conditions of French bulldogs with hemivertebrae in this study (n=141).

| **Disease/condition** | **Hemivertebrae**  **(n=141)** | **Prevalence** | **Confidence Interval (CI)** | **Odds Ratio (OR)** | ***p*-value** |
| --- | --- | --- | --- | --- | --- |
| Spondylosis | 35 | 0.248 | 0.18 – 0.33 | 15.6 | *<*.001*** |
| Osteoarthritis | 27 | 0.191 | 0.14 – 0.26 | 5.8 | *<*.001*** |
| Intervertebral disc disease | 52 | 0.369 | 0.29 – 0.45 | 4.2 | *<*.001*** |
| BOAS | 40 | 0.284 | 0.22 – 0.36 | 4.2 | *<*.001*** |
| Hip dysplasia (HD) | 14 | 0.099 | 0.06 – 0.16 | 3.9 | *<*.001*** |
| Exophthalmos | 14 | 0.099 | 0.06 – 0.16 | 3.9 | *<*.001*** |
| Hypoplastic trachea | 14 | 0.099 | 0.06 – 0.16 | 3.6 | .002** |
| Patellar luxation | 29 | 0.206 | 0.15 – 0.28 | 3.2 | *<*.001*** |
| Elongated soft palate | 63 | 0.447 | 0.37 – 0.53 | 3.1 | *<*.001*** |
| Constricted ear canal | 13 | 0.092 | 0.05 – 0.15 | 2.3 | .022* |
| Stenotic nares | 65 | 0.478 | 0.40 – 0.56 | 2.0 | *<*.001*** |
| Short nose | 64 | 0.454 | 0.37 – 0.54 | 1.9 | *<*.001*** |

** p < 0.05; ** p < 0.01; *** p < 0.001; BOAS: brachycephalic obstructive airway syndrome*

Supplement 13: Reproductive tract disorders in French bulldogs in this study.

| **Disease/condition** | **Occurrences (Fraction)** | **Total Preva- lence** | **Confidence Interval (CI)** | **Female Preva- lence** | **Male Preva- lence** |
| --- | --- | --- | --- | --- | --- |
| Pseudopregnancy | 93/282^1^ | - | 0.28 - 0.39 | 0.33 | - |
| Dystocia | 18/60^2^ | - | 0.20 - 0.43 | 0.30 | - |
| Pyometra | 33/282^1^ | - | 0.08 - 0.16 | 0.12 | - |
| Cryptorchidism | 29/292^3^ | - | 0.07 - 0.14 | - | 0.10 |

**^1^***Only female dogs included. ^2^Only whelping dogs included. ^3^Only male dogs included*

Supplement 14: Summary of the number of litters and puppy counts per litter for all French Bulldog bitches reported to have given birth (n=60).

| **Variable** | **Category** | **Count** |
| --- | --- | --- |
| Number of litters per bitch | One litter | 21 |
|  | Two litters | 13 |
|  | Three litters | 11 |
|  | Four litters | 1 |
|  | Five or more litters | 2 |
| Puppies per litter | 1–2 puppies | 8 |
|  | 3–4 puppies | 18 |
|  | 5–6 puppies | 32 |
|  | 7–8 puppies | 23 |
|  | 9–10 puppies | 7 |
|  | *>*10 puppies | 6 |

Supplement 15: Neurological conditions in the studied population (n=574) and comparison of their prevalences in male *vs.* female dogs.

| **Disease/condition** | **Occurrences** | **Total Preva- lence** | **Confidence Interval (CI)** | **Female Preva- lence** | **Male Preva- lence** | ***p*-value** |
| --- | --- | --- | --- | --- | --- | --- |
| Intervertebral disc disease | 105 | 0.183 | 0.15 – 0.22 | 0.145 | 0.219 | *<*.029* |
| Epilepsy | 21 | 0.037 | 0.02 – 0.06 | 0.035 | 0.038 | 1.000 |
| Dementia | 14 | 0.024 | 0.01 – 0.04 | 0.028 | 0.021 | 0.736 |
| Spinal subarachnoid diverticulum | 5 | 0.009 | 0.00 – 0.02 | 0.007 | 0.010 | 1.000 |
| Meningoencephalitis | 3 | 0.005 | 0.00 – 0.02 | 0.004 | 0.007 | 1.000 |
| Fibrinoid leukodystrophy | 2 | 0.003 | 0.00 – 0.01 | 0.004 | 0.003 | 1.000 |

** p < 0.05; ** p < 0.01; *** p < 0.001*

Supplement 16: Urinary tract disorders in French bulldogs in this study (n=574) and comparison of their prevalences in females vs. male dogs.

| **Disease/condition** | **Occurrences** | **Total Preva- lence** | **Confidence Interval (CI)** | **Female Preva- lence** | **Male Preva- lence** | **p-value** |
| --- | --- | --- | --- | --- | --- | --- |
| Cystitis | 81 | 0.141 | 0.12 - 0.17 | 0.177 | 0.106 | .020* |
| Bladder stones | 15 | 0.026 | 0.02-0.04 | 0.035 | 0.017 | .265 |
| Renal dysplasia | 4 | 0.007 | 0.00 - 0.02 | 0.011 | 0.003 | .591 |
| Chronic kidney disease | 4 | 0.007 | 0.00 - 0.02 | 0.014 | - | .123 |
| Acute renal insufficiency | 3 | 0.005 | 0.00 - 0.02 | 0.007 | 0.003 | .976 |
| Leptospirosis | 1 | 0.002 | 0.00 - 0.01 | - | 0.003 | - |
| Ectopic ureter | 1 | 0.002 | 0.00 - 0.01 | 0.004 | - | - |

** p < 0.05; ** p < 0.01; *** p < 0.001*

Supplement 17: Prevalence of neoplastic conditions in the studied population (n=574) and comparison of the prevalences between male *vs.* female dogs.

| **Disease/condition** | **Occurrences** | **Total Preva- lence** | **Confidence Interval (CI)** | **Female Preva- lence** | **Male Preva- lence** | ***p*-value** |
| --- | --- | --- | --- | --- | --- | --- |
| Mast cell tumor | 26 | 0.045 | 0.03 – 0.07 | 0.057 | 0.034 | 0.274 |
| Mammary tumor | 19 | - | - | 0.064 | - | - |
| Splenic neoplasia | 11 | 0.019 | 0.01 – 0.03 | 0.014 | 0.024 | 0.582 |
| Melanoma | 8 | 0.014 | 0.01 – 0.03 | 0.018 | 0.010 | 0.685 |
| Brain tumor | 7 | 0.012 | 0.01 – 0.02 | 0.014 | 0.010 | 0.963 |
| Hepatic neoplasia | 6 | 0.010 | 0.00 – 0.02 | 0.004 | 0.017 | 0.235 |
| Lymphoma | 5 | 0.009 | 0.00 – 0.02 | 0.011 | 0.007 | 0.969 |
| Cardiac neoplasia | 5 | 0.009 | 0.00 – 0.02 | 0.004 | 0.014 | 0.390 |
| Osteosarcoma | 2 | 0.003 | 0.00 – 0.01 | 0.004 | 0.003 | 1.000 |
| Urinary bladder tumor | 2 | 0.003 | 0.00 – 0.01 | 0.004 | 0.003 | 1.000 |
| Testicular tumor | 2 | - | - | - | 0.007 | - |
| Renal neoplasia | 1 | 0.002 | 0.00 – 0.01 | - | 0.003 | - |
| Insulinoma | 1 | 0.002 | 0.00 – 0.01 | - | 0.003 | - |

Supplement 18: Prevalence of cardiovascular, infectious, and autoimmune diseases in female vs. male French bulldogs in this study (n=574).

| **Disease/condition** | **Occurrences** | **Total Preva- lence** | **Confidence Interval (CI)** | **Female Preva- lence** | **Male Preva- lence** | **p-value** |
| --- | --- | --- | --- | --- | --- | --- |
| Cardiac arrhythmia | 9 | 0.016 | 0.01 – 0.03 | 0.011 | 0.021 | .536 |
| Pulmonary stenosis | 7 | 0.012 | 0.01 – 0.02 | 0.014 | 0.010 | .963 |
| Mitral valve endocardiosis | 6 | 0.010 | 0.00 – 0.02 | 0.007 | 0.014 | .713 |
| Autoimmune disease | 6 | 0.010 | 0.00 – 0.02 | 0.018 | 0.003 | .203 |
| Systemic hypertension | 3 | 0.005 | 0.00 – 0.02 | 0.000 | 0.010 | .259 |
| Leishmaniasis | 3 | 0.005 | 0.00 – 0.02 | 0.004 | 0.007 | 1.000 |
| Heartworm disease | 3 | 0.005 | 0.00 – 0.02 | 0.007 | 0.003 | .976 |
| Ventricular septum defect | 2 | 0.003 | 0.00 – 0.01 | 0.007 | 0.000 | .463 |
| Babesiosis | 2 | 0.003 | 0.00 – 0.01 | 0.000 | 0.007 | .494 |
| Hemophilia | 1 | 0.002 | 0.00 – 0.01 | 0.004 | 0.000 | .986 |
