# Supplementary material for: Health status and disease prevalences in French bulldogs in Germany: insights from a survey-based study
Source: Companion Anim Health Genet. 2025 Oct 31;12:9. doi: 10.1186/s40575-025-00149-8 (PMC12577395; doi:10.1186/s40575-025-00149-8)
Supplement: Supplementary file 3 — Supplementary Material 3. [file 40575_2025_149_MOESM3_ESM.docx]

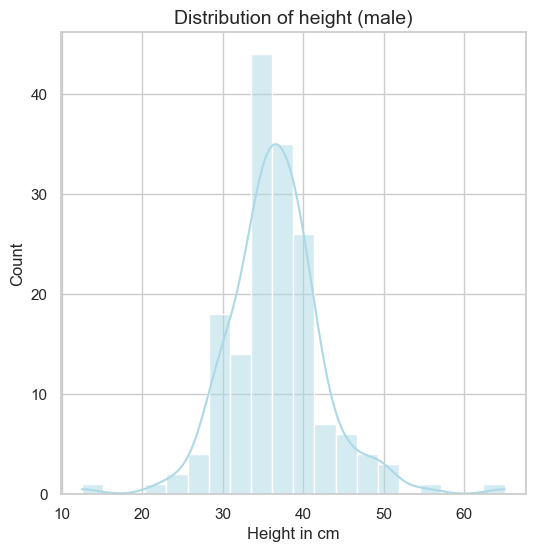


**Supplement 1:** **Height distribution in male French bulldogs >1 year of age**. This histogram displays the frequency distribution of height among male dogs in the dataset. The x-axis indicates height in centimeters, while the y-axis shows the number of individuals per height interval. The overlaid blue line represents a kernel density estimate, providing a smoothed visualization of the height distribution.


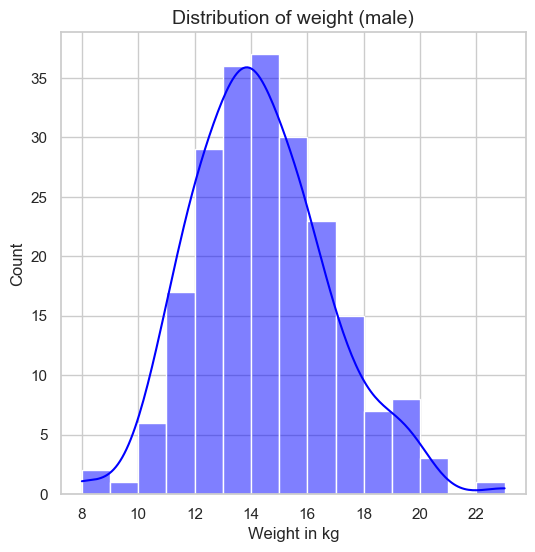


**Supplement 2**: **Weight distribution in male French bulldogs >1 year of age**. This histogram displays the frequency distribution of body weight among male dogs in the dataset. The x-axis indicates weight in kilograms, while the y-axis shows the number of individuals per weight interval. The overlaid blue line represents a kernel density estimate, providing a smoothed visualization of the weight distribution.


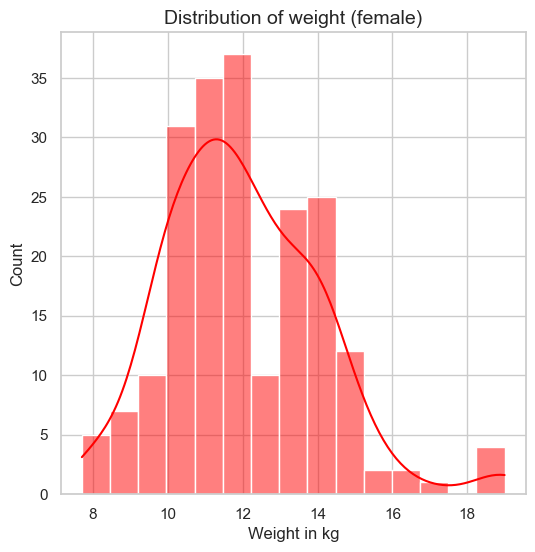


**Supplement 3:** **Weight distribution in female French bulldogs >1 year of age**. This histogram displays the frequency distribution of body weight among female dogs in the dataset. The x-axis indicates weight in kilograms, while the y-axis shows the number of individuals per weight interval. The overlaid blue line represents a kernel density estimate, providing a smoothed visualization of the weight distribution.


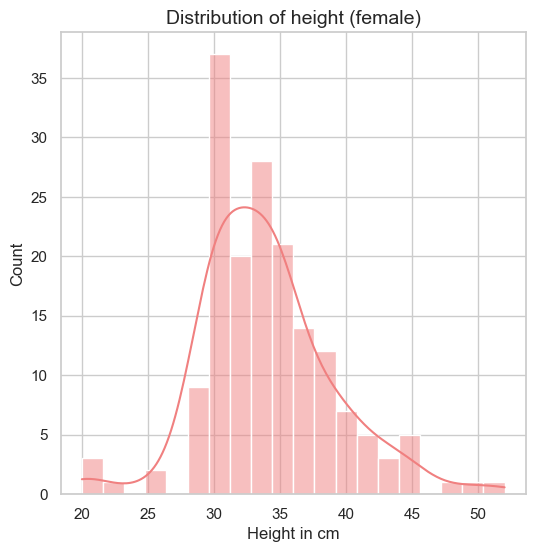


**Supplement 4:** **Height distribution in female French bulldogs >1 year of age.** This histogram displays the frequency distribution of height among female dogs in the dataset. The x-axis indicates height in centimeters, while the y-axis shows the number of individuals per height interval. The overlaid blue line represents a kernel density estimate, providing a smoothed visualization of the height distribution.
